# DUB3 Deubiquitylating Enzymes Regulate Hippo Pathway Activity by Regulating the Stability of ITCH, LATS and AMOT Proteins

**DOI:** 10.1371/journal.pone.0169587

**Published:** 2017-01-06

**Authors:** Hung Thanh Nguyen, Jan-Michael Kugler, Stephen M. Cohen

**Affiliations:** Department of Cellular and Molecular Medicine, University of Copenhagen, Copenhagen, Denmark; Wayne State University, UNITED STATES

## Abstract

The YAP and TAZ transcriptional coactivators promote oncogenic transformation. Elevated YAP/TAZ activity has been documented in human tumors. YAP and TAZ are negatively regulated by the Hippo tumor suppressor pathway. The activity and stability of several Hippo pathway components, including YAP/TAZ, is regulated by ubiquitin mediated protein turnover and several ubiquitin ligase complexes have been implicated in human cancer. However, little is known about the deubiquitylating enzymes that counteract these ubiquitin ligases in regulation of the Hippo pathway. Here we identify the DUB3 family deubiquitylating enzymes as regulators of Hippo pathway activity. We provide evidence that DUB3 proteins regulate YAP/TAZ activity by controlling the stability of the E3 ligase ITCH, the LATS kinases and the AMOT family proteins. As a novel Hippo pathway regulator, DUB3 has the potential to act a tumor suppressor by limiting YAP activity.

## Introduction

Transcriptional co-activators Yes-Associated Protein 1 (YAP1) and transcriptional coactivator with PDZ-binding motif (TAZ) mediate the activity of the Hippo signaling pathway in control of cell proliferation and in tumor progression [1]. YAP and TAZ bind to a number of different transcription factors to regulate genes required for cell proliferation and survival [2]. Increases in YAP/TAZ expression by epigenetic regulation and gene amplification have been observed in human cancers, as have mutations in the upstream elements of the Hippo pathway that alleviate Hippo mediated repression of YAP/TAZ activity [3–5]. More recently, increased YAP activity has been shown to be sufficient to replace the requirement for oncogenic K-Ras in models of pancreatic and colon cancer [6,7], and in the transformation of primary human cells to create cancer cells [8,9]. By limiting YAP/TAZ activity, the Hippo pathway serves as a barrier to cellular transformation and tumor formation.

A number of different mechanisms have been reported by which ubiquitin-mediated protein turnover regulates YAP/TAZ activity. The best studied of these involves the targeting of YAP and TAZ for destruction by the βTrCP/SCF ubiquitin ligase system, as a consequence of LATS1/2 mediated phosphorylation of YAP and TAZ [10,11]. Destruction of YAP and TAZ contributes to the tumor suppressive effects of the Hippo signaling pathway. Conversely, the transforming potential of oncogenic Ras is mediated in part through promoting YAP/TAZ stability. Ras acts by downregulating the expression of the substrate recognition factors that recruit YAP to an Elongin B/C-Cullin5 ubiquitin ligase complex [8]. Ubiquitin-mediated protein turnover is involved in regulating the activity of a number of other Hippo pathway components. Mechanisms for regulation of LATS kinase activity and turnover have been reported. The E3 ubiquitin ligases ITCH and NEDD4 have been shown to promote turnover of LATS kinases, and ITCH upregulation can promote tumorigenesis through increased YAP/TAZ activity [12] [13,14]. The RING ligase PRAJA2 promotes turnover of MOB1, a regulator of LATS kinases, and has been implicated in glioblastoma [15]. Other YAP/TAZ regulators are also controlled by ubiquitylation. Angiomotin (AMOT) and the related AMOT-like proteins interact with both LATS kinases and with YAP. AMOT proteins inhibit YAP/TAZ activity through direct physical association and by promoting LATS-mediated phosphorylation of YAP [16,17]. AMOT turnover is regulated by ITCH and other Nedd4 ubiquitin ligase family members [18]. Thus, ubiquitin-based protein turnover acts at multiple levels in the core Hippo pathway as well as in ancillary pathways to control YAP/TAZ stability and activity.

The activity of ubiquitin ligases in promoting protein turnover can be counteracted by deubiquitylating enzymes that remove ubiquitin moieties from proteins. Evidence is emerging that deubiquitylating enzymes may act as oncogenes and tumor suppressors [19–22]. Using a cell-based RNAi screen for YAP/TAZ activity, we recently identified the deubiquitylating enzyme USP9x as a negative regulator of YAP/TAZ activity, acting through regulation of AMOT turnover [23]. These findings provide a molecular framework for the observation that low USP9x expression correlated with poor survival of renal clear cell carcinoma [23] and that USP9x is downregulated in pancreatic ductal adenocarcinoma [22].

In this report, we present evidence implicating the family of DUB3 deubiquitylating enzymes in control of Yap/TAZ activity. DUB3 proteins act by regulating the stability of the E3 ubiquitin ligase ITCH, the Hippo pathway core kinases LATS1 and LATS2, as well as the family of AMOT proteins, which play a scaffolding role in DUB3-mediated regulation of YAP.

## Materials and Methods

### Antibodies

Antibodies to NEDD4 (Cat #2740), ITCH (#12117), SMURF1 (#2174), phosphor-YAP (S127, #4911), YAP (#4912), YAP/TAZ (#8418), phospho-LATS1 (T1079,#8654), LATS1 (#9153), LATS2 (#13646), specific K48 and K63 ubiquitin and Myc Tag (#2278) were from Cell Signaling Technology (Danvers, MA, USA). HA (#sc-7392) and YAP (sc#101199) antibodies were from Santa Cruz Biotechnology (Dallas, TX, USA). Anti-AMOT was from ABNOVA (Taipei, Taiwan). Anti-Flag antibody and anti-Actin, HA-, Myc- and Flag-conjugated beads were from Sigma-Aldrich, St Louis, MO, USA). DUB3 antibody (Cat#PA-5-44961) was obtained from Invitrogen (Taastrup, Denmark).

### Plasmids and siRNAs

The DUB shRNA library was described in [23]. 8xGTIIC-luciferase was a gift from Stefano Piccolo (Addgene plasmid # 34615). The pRL-CMV (Renilla, #E2261) was purchased from Promega (Madison, WI, USA). pBabe HA-LATS2 and HA-p130 AMOT expressing plasmids were kind gifts from SW Chan (IMCB). Flag-DUB3 was cloned from cDNA of 293T cells by PCR into a pBabe (puro) expression vector using the following primers: ATACGGATCCACCATGGACTACAAGGATGACGATGACAAGGACTACAAGGATGACGATGACAAGGAGGACGACTCACTCTACTT and ATCAGAATTCTCACTGGCACACAAGCAGAG. The PCR product was sequenced and encodes the USP17L17 isoform of DUB3. The C89S mutant was made by PCR as previously described [24], and confirmed by sequencing. LATS2 shRNA was described in [25]. The pBabe vector expressing Myc-ITCH was subcloned from the PCINeo-myc-ITCH plasmid, which was a gift from Allan Weissman (Addgene plasmid # 11427). Single and smart pool siRNAs against LATS2, AMOT, AMOT L1, AMOT L2, ITCH, NEDD4 and DUB3 were purchased from GE Dharmacon (Brøndby, Denmark). Details of the siRNA sequences used to deplete DUB3 family members are provided in S1 Table.

### Luciferase assays

Luciferase assay to measure YAP/TAZ activity were performed as described [23] using a dual luciferase kit (E1960, Promega).

### Quantitative real-time RT-PCR

RNA was extracted in Trizol (Invitrogen) and pretreated with DNAse (RQ1, Promega) before being used for cDNA synthesis. cDNA was made using iScript (BioRad) with random hexamer primers. At least two pairs of qPCR primers were tested for specificity and sensitivity for each mRNA. Real-time PCR used SyberGreen I Mix on a QuantStudio 6 Flex machine (ABI, Thermo Fisher Scientific, Waltham, MA, USA). As DUB3 is an intron-less gene, a parallel analysis of non-iScript and iScript treated cDNA was performed to exclude the possibility of amplification from genomic DNA-contaminated samples. Primer sequences are in S1 Table.

### Cell culture, transfection, immunoprecipitation and blotting

BJ foreskin fibroblasts and HEK293T cells were obtained from ATCC and cultured in DMEM (Sigma) with 10% fetal calf serum (HyClone) and 1% penicillin-streptomycin. HEK293T cells were transfected with using the Calcium phosphate method. BJ fibroblast cells were transfected using Fugene HD (Promega) according the manufacturer’s instructions. For immunoblotting, transfected cells were washed with cold TBS after 48h and lysed in modified RIPA buffer containing 25mM Tris/HCl, pH 7.5, 150mM NaCl, 0.5% Nonidet NP40, 0.1% Na-deoxycholate, 1mM EDTA, 0.5% Triton X-100, 5mM NaF, 5mM β-glycerophosphate, 1mM Na_3_VO_4_ and protease inhibitor cocktail. After incubation with antibody, blots were developed with Western Lightning Plus-ECL reagent (PerkinElmer). For co-immunoprecipitation assays, cells were pretreated with 5μM MG132 overnight before being harvested for immunoprecipitation. Transfected cells were washed once with cold TBS (20mM Tris-HCl pH 7.5, 150mM NaCl) and lysed with PLC buffer [26] containing 50 mM HEPES pH 7.5, 150 mM NaCl, 5% Glycerol, 0.5% Triton X-100, 1.5 mM MgCl2, 1 mM EGTA supplemented with 20 μg/ml RNAase A, 1 mM DTT, 1 mM Na_3_VO_4_ and protease inhibitor cocktail. Cell lysates were pre-cleared with proteins A/G beads (Santa Cruz, sc#2003) and immunoprecipitated for 2 hours at 4°C with anti-HA, or–Myc or -Flag agarose beads. Beads were washed 4x with lysis buffer and eluted as recommended by the manufacturer. For ubiquitin assays, a minimal amount of ITCH expression plasmid (3μg) was used to co-transfect with 2μg of ubiquitin expression plasmids in 10-cm disc of HEK293T cells and the PLC lysis buffer was freshly supplemented with 10mM of N-ethylmaleimide (NEM, Sigma).

### Statistical analyses

Differences among conditions and treatments for luciferase assays, quantitative PCR and cell growth assays were determined using the Student’s t-test (2-tailed, unequal variance). For assessing differences in subcellular localization of YAP localization, the Chi-square test was used.

## Results and Discussion

### DUB3 regulates YAP activity

The DUB3 family of deubiquitylating enzymes was identified in a shRNA-based screen for regulators of YAP/TAZ activity [23]. The DUB3 family includes 25 USP17-like proteins. These proteins are encoded, in varying numbers, in tandem repetitive sequences in human genomes [27,28]. Sequence divergence among the members of this family is low, however, no single siRNA or shRNA sequence targets all family members, nor are there reagents that allow selective targeting of only one family member (S1 Fig). Quantitative PCR and immunoblot analysis showed that the shRNAs and siRNA pools used in this study were effective in depleting the family of DUB3 transcripts and proteins (S2 Fig).

**Fig 1:**
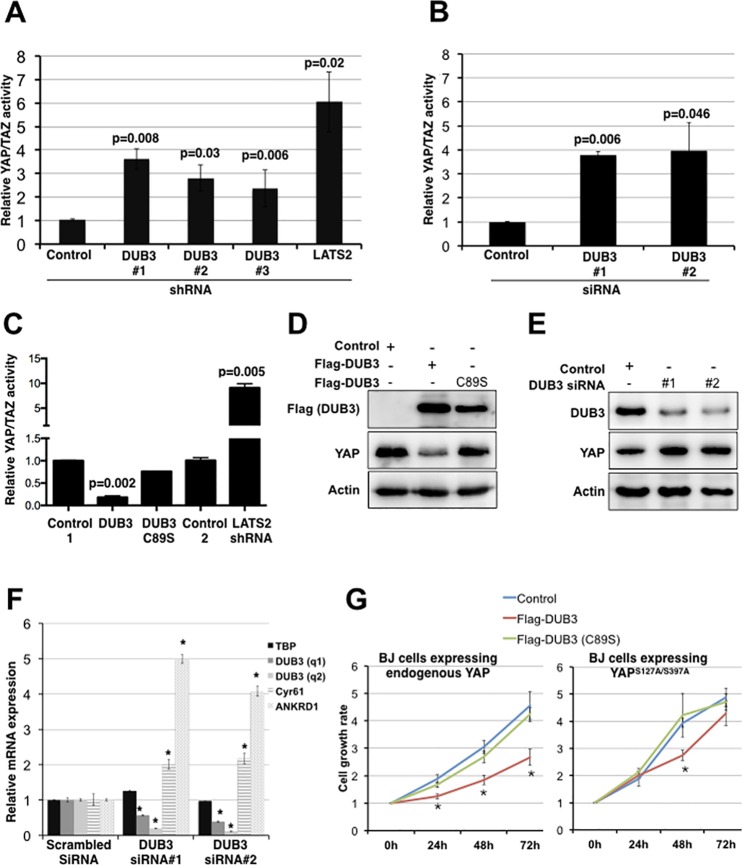
DUB3 regulates Hippo activity by mediating YAP turnover. (A) Luciferase reporter assays showing the effects of DUB3 shRNAs on YAP/TAZ activity. HEK293T cells were transfected to express the 8XGTIIC_luc YAP/TAZ reporter, which contains 8 TEAD binding sites to control the expression of firefly luciferase and a vector expressing CMV-Renilla luciferase to normalize for transfection efficiency, together with independent shRNA vectors to deplete DUB3 or with a control shRNA. shRNA to deplete LATS2 was used as a positive control. Data represent the average of three independent transfection experiments ± SD. P values were determined using Student’s T-test (2-tailed, unequal variance). (B) Luciferase reporter assays showing the effects of DUB3 siRNAs on YAP/TAZ activity. HEK293T cells were transfected to express the luciferase reporters together with independent siRNAs to deplete DUB3 or with a control scrambled siRNA. Data represent the average of three independent transfection experiments ± SD. P values were determined using Student’s T-test (2-tailed, unequal variance). (C) Luciferase reporter assays showing the effect of DUB3 on YAP/TAZ activity. HEK293T cells were transfected to express the luciferase reporters together with a vector expressing Flag-DUB3, the C89S mutant form of DUB3 or relevant controls vectors. shRNA to deplete LATS2 was used as a positive control. Data represent the average of three independent replicates ± SD. P values were determined using Student’s T-test (2-tailed, unequal variance). (D) Immunoblots showing the effect of DUB3 expression on YAP protein. HEK293T cells were transfected with a vector expressing Flag-DUB3, the C89S mutant form of DUB3 or a control vector. Blots were probed with anti-YAP antibody and anti-Flag. Anti-Actin was used to control for loading. (E) Immunoblots showing the effect of DUB3 depletion on the YAP expression level. HEK293T cells were transfected with independent DUB3 siRNAs or a scrambled siRNA control. Blots were probed with anti-YAP antibody and anti-Flag. Anti-Actin was used to control for loading. (F) Effect of DUB3 siRNAs on the expression of YAP transcriptional targets. HEK293T cells were transfected with DUB3 or control siRNAs. mRNA expression of DUB3, Cyr61, ANKRD1 was measured by RT-PCR. GAPDH mRNA was used for normalization and TBP was used as an additional control gene. Data represent the average of 3 independent experiments ± SD. * indicates p <0.01, compared to the relevant controls (Student’s T-test; 2-tailed, unequal variance). (G) Effect of DUB3 on cell growth. Human primary fibroblast BJ cells were engineered to express hTert, H-Ras^G12V^ and to deplete p53 and p16, or with the addition or YAP^S127A/S397A^. BJ^p53kd/p16kd/HRas^ and BJ^p53kd/p16kd/HRas/YAPS127A/S397A^ cells were virally transduced and selected to stably express DUB3, its inactive C89S mutant form or with an empty vector as a control. Cells were counted at 24-hour intervals for a period of 72h. The assay was performed in triplicate. Data represent the average ± SD. * indicate p <0.05, compared to the relevant controls (Student’s T-test; 2-tailed, unequal variance).

Depletion of DUB3 transcripts by three independent shRNAs significantly increased YAP/TAZ reporter activity (Fig 1A). Depletion of LATS2 was used as a positive control for increased YAP/TAZ activity in this assay. This was verified using two additional pools of siRNAs to deplete DUB3 (Fig 1B; controls are in S2 Fig). Reciprocally, overexpression of DUB3 led to a significant reduction of YAP reporter activity (Fig 1C). Expression of a catalytically inactive mutant form of DUB3 (C89S, [29]) had no effect on YAP luciferase reporter activity (Fig 1C), indicating that the enzymatic activity of DUB3 is required for its effects on YAP activity.

Immunoblotting of lysates from cells overexpressing DUB3 showed a marked decrease in the level of YAP protein, consistent with the decrease in the luciferase reporter assay (Fig 1D; YAP mRNA levels were not affected, S3 Fig). The catalytically inactive C89S form of DUB3 had no effect on YAP levels (Fig 1D). Reciprocally, depletion of DUB3 proteins with two independent siRNA pools led to an increase in YAP protein levels (Fig 1E) and to a significant increase in expression of YAP transcriptional targets Cyr61 and ANKRD1 (Fig 1F). Comparable results were obtained by depleting DUB3 in BJ cells (S4 Fig). Nuclear localization of YAP increased in BJ cells depleted of DUB3 compared to control cells, consistent with the observed increase in YAP reporter activity and target gene expression (S4 Fig and S5 Fig). These data indicate that DUB3 regulates the abundance of YAP protein, its subcellular localization and the expression of its targets.

### DUB3 inhibits cell growth through regulating Hippo pathway activity

YAP acts through TEAD proteins to regulate a number of targets that promote cell growth and proliferation [30]. Our results showed that DUB3 inhibits YAP/TAZ activity; we asked whether this inhibition would affect Hippo-mediated cell growth and proliferation. To investigate this, we utilized human primary fibroblast BJ cells (BJ^p53kd/p16kd/HRas^), in which cell growth is affected by modulating Hippo activity [9]. Co-expression of DUB3 significantly reduced growth of these cells, compared to cells expressing the catalytically inactive C89S mutant form of DUB3 or the control vector (Fig 1G). DUB3 expression had a more limited effect on the growth of BJ cells expressing a mutant form of YAP (S127A/S397A) that is not regulated by LATS kinases (BJ^p53kd/p16kd/HRas/YAPS127A/S397A^; Fig 1G). The residual effect of DUB3 may be due to the endogenous wild-type YAP that is also expressed in these cells.

### DUB3 acts via the E3 ubiquitin ligase ITCH

As a deubiquitylating enzyme, overexpression of DUB3 is expected to remove ubiquitin from its direct targets, thereby increasing their stability. However, we observed that overexpression of DUB3 led to a decrease in YAP protein levels. Conversely, depletion of DUB3 should increase ubiquitylation, leading to a decrease in target protein levels. Instead, DUB3 depletion increased YAP protein levels. DUB3 is therefore unlikely to act directly on YAP, so we tested other regulators of YAP/TAZ activity to identify potential direct targets of DUB3.

**Fig 2:**
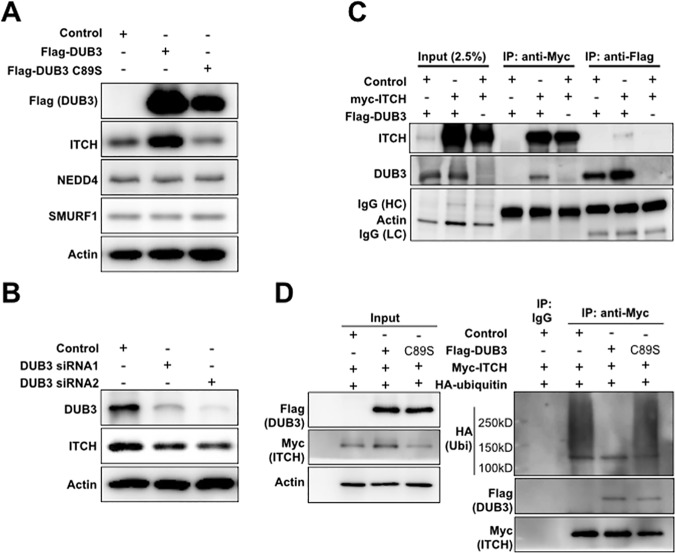
DUB3 interacts with ITCH and mediates its stability. (A) Immunoblots showing the effect of DUB3 expression on ITCH. HEK293T cells were transfected with a vector expressing Flag-DUB3, its C89S DUB3 or a control vector. Blots were probed with anti-Flag, anti-ITCH, anti-NEDD4 and anti-SMURF1 antibodies. Anti-Actin was used to control for loading. (B) Immunoblots showing the effect of DUB3 siRNAs on ITCH. HEK293T cells were transfected with independent siRNAs against DUB3 or a scrambled siRNA. Blots were probed with antibodies against ITCH or Actin for loading control. (C) Immunoprecipitation assays showing interaction between DUB3 and ITCH. HEK293T cells were transfected to express Flag-tagged DUB3 and Myc-tagged ITCH as indicated. Transfected cells were treated with MG132 5μM overnight before being harvested for immunoprecipitation with anti-Flag or anti-Myc-conjugated beads. Blots were probed with anti- Flag to detect DUB3 or anti-Myc to detect ITCH. (D) Ubiquitylation assay showing the effect of DUB3 on ITCH ubiquitylation. HEK293T cells were co-transfected with a vector expressing Myc-ITCH and a vector expressing Flag-tagged DUB3, Flag-tagged DUB3 C89S or a control vector. Transfected cells were treated with MG132 5μM overnight before being harvested for immunoprecipitation with anti-Myc-or isotype IgG-conjugated beads in PLC buffer freshly supplemented with 10mM of NEM. Immunoblots were probed with antibodies against HA, Flag and Myc.

Deubiquitylating enzymes have been reported to associate with ubiquitin E3 ligases and to regulate their stability and activity [31]. We asked whether DUB3 overexpression affected the expression of the E3 ligases known to regulate the Hippo pathway. ITCH and NEDD4 have been implicated in turnover of LATS kinases and AMOT [12] [13,14,18]. Overexpression of DUB3 led to a marked increase in ITCH protein, but had little or no effect on the amount of NEDD4 or SMURF1 (Fig 2A). Expression of the catalytically inactive C98S form of DUB3 had no significant effect on ITCH levels. Quantitative PCR showed no change in the levels of the ITCH, NEDD4 or SMURF1 transcripts (S6 Fig). Reciprocally, siRNA-mediated depletion of DUB3 proteins led to a decrease in ITCH protein levels (Fig 2B).

To investigate the possibility that DUB3 might act directly on ITCH, we tested for physical interaction. Flag-tagged DUB3 was coexpressed with Myc-tagged ITCH. Lysates were immunoprecipitated with anti-Myc and probed with anti-Flag to detect DUB3. DUB3 was recovered by co-precipitation with ITCH (Fig 2C). Likewise, ITCH was recovered by co-precipitation with Flag-DUB3 (Fig 2C). Endogenous ITCH was pulled down by co-immunoprecipitation with DUB3, but was not recovered in the control IgG immunoprecipitates (S7 Fig). These data suggest that DUB3 physically interacts with ITCH.

Next, we asked whether DUB3 affects ubiquitylation of ITCH. Acting as an E3 ubiquitin ligase, ITCH can auto-ubiquitylate in an intermolecular manner [32] [33]. The USP9x deubiquitylating enzyme has been shown to promote ITCH stability by counteracting auto-ubiquitylation [32]. To monitor ITCH ubiquitylation, cells were transfected to express Myc-tagged ITCH along with HA-tagged Ubiquitin and with Flag-tagged DUB3 or with a control vector. Cells were incubated in the presence of the proteasome inhibitor MG132 to limit degradation of ubiquitylated proteins. Myc-tagged ITCH was then recovered by immunoprecipitation and the blots were probed with anti-HA to detect HA-tagged ubiquitin and with anti-Flag to detect Flag-tagged DUB3. Expression of DUB3 strongly reduced the amount of poly-ubiquitylated ITCH (Fig 2D). Expression of the inactive C89S mutant form of DUB3 had little or no effect on ITCH ubiquitylation (Fig 2D). Reciprocally, depletion of DUB3 proteins led to an increase in ITCH ubiquitylation (S8 Fig). These results suggest that DUB3 modulates ubiquitylation of ITCH and thereby regulates its stability by protecting it from degradation.

### DUB3 stabilizes LATS and AMOT proteins

**Fig 3:**
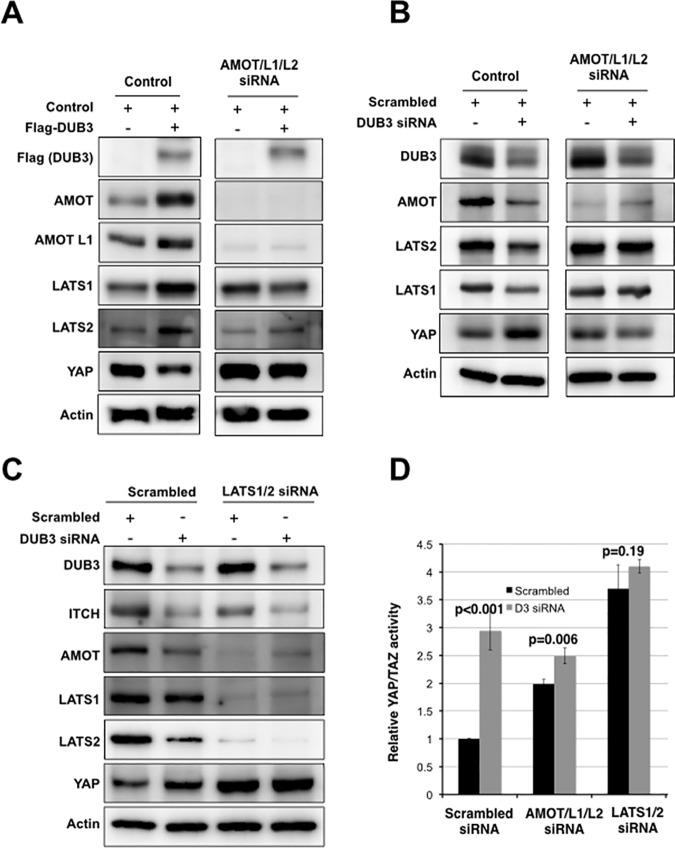
LATS and AMOT proteins are required for DUB3-mediated regulation of Hippo signaling. (A) Immunoblots showing the effect of DUB3 expression on LATS kinases, AMOT and YAP. HEK293T cells were transfected with a vector expressing Flag-DUB3 or a control vector together with a mixture of siRNAs targeting AMOT, AMOTL1 and AMOTL2 or a scrambled siRNA control. Blots were probed with antibodies against Flag, AMOT, AMOTL1, LATS1, LATS2 and YAP. Anti-Actin was used to control for loading. Samples were run on the same SDS-acrylamide gels with intervening lanes removed. (B) Immunoblots showing the effect of DUB3 depletion on LATS kinases, AMOT and YAP. HEK293T cells were transfected with a vector expressing Flag-DUB3 or a control vector together with a mixture of siRNAs targeting AMOT, AMOTL1 and AMOTL2 or a scrambled siRNA control. Blots were probed with antibodies against DUB3, AMOT, LATS1, LATS2 and YAP. Anti-Actin was used to control for loading. Samples were run on the same gels with intervening lanes removed. (C) Immunoblots showing the effect of DUB3 depletion on ITCH, LATS kinases and AMOT. HEK293T cells were transfected with a control or DUB3 siRNA in the presence of LATS1 and LATS2 siRNAs or a scrambled siRNA control. Blots were probed with antibodies against DUB3, ITCH, AMOT, LATS1, LATS2 and YAP. Anti-Actin was used to control for loading. (D) Luciferase reporter assays showing the effects of DUB3 siRNAs on YAP/TAZ activity. HEK293T cells were transfected to express the luciferase reporters together with a control or DUB3 siRNA in the presence of a mixture of siRNAs targeting AMOT, AMOTL1 and AMOTL2, a mixture of siRNAs targeting LATS1 and LATS2 or a scrambled siRNA control. Data represent the average of three independent transfection experiments ± SD. P values were determined using Student’s T-test (2-tailed, unequal variance).

ITCH-mediated ubiquitylation has been shown to promote the turnover of several Hippo components, such as LATS kinases and AMOT proteins [12,18,34]. Our results suggest that DUB3 promotes ITCH stability; we therefore asked if DUB3 would affect the stability of these Hippo proteins as well. Interestingly, DUB3 expression increased the levels of AMOT, AMOTL1, LATS1 and LATS2 and a decrease in YAP level. (Fig 3A; transcript levels were unaffected, S3 Fig). Depletion of DUB3 led to a marked decrease in LATS1, LATS2 and AMOT levels (Fig 3B). Decreased LATS levels are consistent with an increase in YAP levels (Fig 3B) and with the observed effect on YAP reporter activity (Fig 1B).

These findings present an apparent conundrum: DUB3 interacts with ITCH and affects ITCH ubiquitylation and stability, and higher ITCH expression should increase turnover of LATS and AMOT. However, despite the increase in ITCH protein, LATS and AMOT were stabilized as a consequence of DUB3 expression. Reciprocally, they were destabilized as a consequence of DUB3 depletion. We therefore considered the possibility that DUB3 might directly regulate the turnover of AMOT and LATS proteins.

We examined the effect of DUB3 depletion on AMOT ubiquitylation. In control cells, DUB3 was recovered by co-precipitation with AMOT, providing evidence for physical interaction (S9 Fig). Depletion of DUB3 led to a marked increase of AMOT ubiquitylation (S9 Fig). Reciprocally, overexpression of DUB3 reduced the ubiquitylation of AMOT, while the inactive C89S DUB3 mutant had no effect (S9 Fig). Likewise, we examined the interaction between DUB3 and LATS2. LATS2 was recovered by co-immunoprecipitation of DUB3 (S10 Fig). LATS2 ubiquitylation was markedly suppressed by overexpression of DUB3, but not by the catalytically inactive C89S form of DUB3 (S10 Fig). These data provide evidence that DUB3 interacts with AMOT and LATS2 proteins and regulates their turnover.

### AMOT and LATS1/2 are required for the effect of DUB3 on YAP

AMOT proteins have been proposed to play a scaffolding role, mediating the interaction between ITCH and YAP, to promote YAP ubiquitylation [35]. AMOT proteins also inhibit YAP/TAZ activity through direct physical association and by promoting LATS-mediated phosphorylation of YAP [16,17]. In light of this, we asked if AMOT and the related AMOTL1 and AMOTL2 proteins are required to mediate the effect of DUB3 on LATS kinase and YAP levels. In cells depleted of AMOT, AMOTL1 and AMOTL2, we observed no stabilization of LATS1 or LATS2 proteins following DUB3 overexpression (Fig 3A). The decrease in YAP protein levels caused by DUB3 expression was also lost in the AMOT-depleted cells (Fig 3A). Likewise, the effects of depleting DUB3 on LATS1/2 and YAP protein levels were lost in cells depleted of the AMOT family proteins (Fig 3B). Thus AMOT proteins are required for DUB3 to act on LATS2 and YAP protein levels. Consistent with this, the effects of DUB3 siRNA on YAP reporter activity were significantly blunted by depletion of AMOT proteins (Fig 3D).

Next we asked whether depleting the LATS1/2 kinases would block the effects of DUB3 on YAP levels. DUB3 depletion led to an increase in YAP levels (Fig 3C). Depletion of LATS1/2 kinases led to an increase in the basal level of YAP protein, but there was no further effect of DUB3 on YAP (Fig 3C). However, LATS1/2 depletion did not prevent the effects of DUB3 on ITCH (Fig 3C). We also noted that depletion of LATS1/2 resulted in loss of AMOT protein (Fig 3C). LATS-mediated phosphorylation has previously been reported to stabilize AMOT [36], so loss of AMOT is an expected consequence of LATS1/2 depletion. This experiment provides evidence that LATS1/2 proteins are required to mediate the effects of DUB3 on regulation of YAP activity. This is likely to occur through both through regulating AMOT turnover as well as through their direct phosphorylation of YAP. Consistent with this, the effects of DUB3 on YAP reporter activity were significantly blunted by depletion of LATS1/2 proteins (Fig 3D).

**Fig 4:**
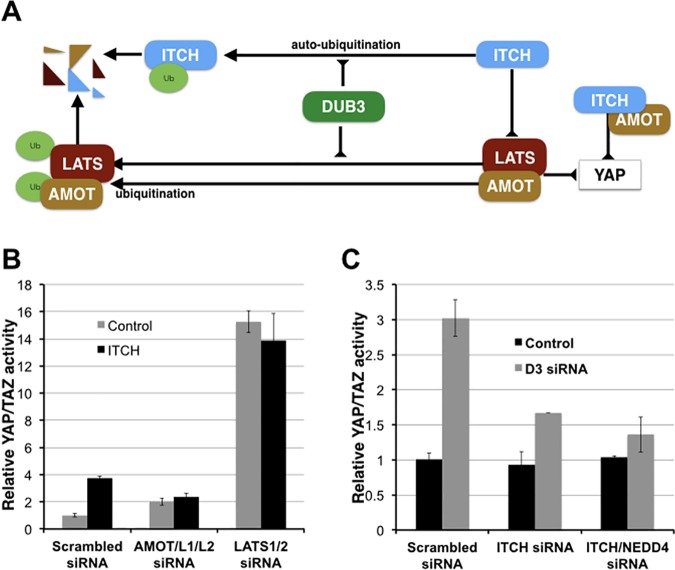
DUB3 mediates ITCH, LATS1/2 and AMOT proteins to regulate Hippo activity. (A) A schematic view of DUB3-mediated regulation of Hippo signaling. DUB3 de-ubiquitylates ITCH, LATS1/2 and AMOT to promote their stability. In the presence of stabilized AMOT, ITCH promotes YAP degradation. (B) Luciferase reporter assays showing the effects of ITCH overexpression on YAP/TAZ activity. HEK293T cells were transfected to express the luciferase reporters together with a control or ITCH expression vector in the presence of a mixture of siRNAs targeting AMOT, AMOTL1 and AMOTL2, a mixture siRNAs targeting LATS1 and LATS2 or a scrambled siRNA control. Data represent the average of three independent transfection experiments ± SD. (C) Luciferase reporter assays showing the effects of DUB3 siRNAs on YAP/TAZ activity. HEK293T cells were transfected to express the luciferase reporters together with a control or DUB3 siRNA in the presence of ITCH siRNA, a mixture of siRNAs targeting ITCH and NEDD4 or a scrambled siRNA control. Data represent the average of three independent transfection experiments ± SD.

Our findings provide evidence that the DUB3 deubiquitylating enzyme acts at several levels to regulate the activity of the Hippo tumor suppressor pathway (Illustrated in Fig 4A). We have identified the E3 Ligase ITCH, LATS1/2 and AMOT as targets for the deubiquitylating activity of DUB3. Each of these proteins serves as regulators of YAP activity.

AMOT proteins have been proposed to provide a platform linking the E3 ligase ITCH and YAP [35]. We confirmed this relationship using the YAP luciferase reporter assay, comparing the effects of ITCH expression in cells depleted of AMOT, AMOTL1 and AMOTL2 with control cells. ITCH expression increased YAP/TAZ activity in control cells, but had no effect in cells lacking the AMOT proteins (Fig 4B; note that the basal level of YAP activity was higher in AMOT-depleted cells). Similarly, removing LATS kinase activity prevented ITCH from increasing YAP activity (Fig 4B). Note that AMOT protein was lost in cells depleted of LATS kinases, so we hypothesize that the effect of either siRNA treatment would be to eliminate the scaffold linking ITCH and YAP (Fig 4A). LATS kinases also phosphorylate YAP and TAZ and target them for destruction by the βTrCP/SCF ubiquitin ligase system [10,11]. By helping to stabilize AMOT, the LATS kinases also promote interaction of ITCH with YAP/TAZ, providing another means to promote YAP/TAZ turnover.

DUB3 limits YAP activity by stabilizing the ubiquitin E3 ligase ITCH concurrently with stabilization of LATS kinases and AMOT proteins. The effects of DUB3 on YAP depend on the presence of AMOT proteins, suggesting that this activity occurs in the context of a protein complex containing the E3 ligase and its substrates. Whether DUB3 is a stable member of this complex containing ITCH, AMOT and LATS kinases is uncertain. DUB3 binds effectively to ITCH, but its association with LATS or AMOT appeared to be less stable in co-IP experiments.

We favor a model in which inhibition of Hippo signaling by DUB3 reflects contributions of DUB3 in stabilizing the AMOT proteins and LATS1/2. Although DUB3 also stabilizes the E3 ligase ITCH, interaction of DUB3 with LATS1/2 and AMOT in this complex is hypothesized to offset the effects of increased ITCH activity toward these substrates. On the other hand, the increased expression of ITCH may facilitate interaction with YAP. Thus DUB3 activity can promote the stability of LATS1/2 and AMOT, which act to reduce YAP activity and increase YAP turnover, while also increasing expression of ITCH, which can promote YAP turnover. Depletion of ITCH from this complex reduces the effects of DUB3 on YAP, and this effect was stronger when NEDD4, another E3 ligase was also removed (Fig 4C). This suggests that multiple E3 ligases can serve in this complex to regulate YAP. Similarly, any of the three AMOT proteins appears to support the activity of DUB3 on YAP activity.

Evidence for links between DUB3 family members and cancer remain equivocal. One study has provided evidence that a DUB3 family might serve as an oncogene by stabilizing CDC25A and promoting cell cycle progression in breast cancer [37]. In another study the DUB3 paralog, USP17 was reported to limit cell cycle progression by inhibiting RAS activity [38]. Our findings suggest that members of the DUB3 family have the potential to act as tumor suppressors by limiting YAP activity. However, given that there are multiple genes encoding DUB3 family members, at distinct chromosomal locations, it is unlikely that this activity will be uncovered by loss of function mutations in human cancer.

## Supporting Information

S1 Fig Summary of DUB3 family members. The NCBI RefSeq Gene collection comprises 30 USP17L (USP17L1 through USP17L30) gene entries: 25 are annotated as protein coding genes (each with an individual mRNA and protein RefSeq), while four are annotated as pseudogenes (USP17L6P, -9P, -14P, -16P), and one (USP17L23) is annotated as protein coding gene, but not linked to either a RefSeq mRNA or protein sequence. (http://www.ncbi.nlm.nih.gov/books/NBK21091). The table summarizes the number of mismatches between the individual siRNAs and shRNAs with the family members.
(PDF)

S2 Fig Effect of shRNAs and siRNAs on DUB3 mRNA expression. Controls for the efficacy of shRNAs and siRNAs on DUB3 mRNA expression. (A) HEK293T cells were transfected with a control vector or with independent shRNAs targeting DUB3. (B) HEK293T cells were transfected with a control siRNA (scrambled sequence) or independent siRNAs targeting DUB3. Expression of DUB3 mRNA was measured by quantitative real-time RT-PCR (qPCR) using two independent pairs of primers. Primer pair #1 was used subsequently for measuring DUB3 expression. GAPDH mRNA was used for normalization. Data represent the average of 3 independent experiments ± SD. * indicates p <0.05 (Student’s T-test, 2-tailed unequal variance) comparing the test and control samples.
(PDF)

S3 Fig controls for the effects of DUB3 depletion. HEK293T cells were transfected with a vector expressing DUB3 or the catalytically-inactive C89S mutant form or with an empty vector as a control. mRNA expression of YAP, TAZ, ITCH, AMOT, AMOT L1, AMOT L2, LATS1, LATS2 and DUB3 was measured by qPCR. GAPDH mRNA was used as a normalization standard and TBP was used as an independent control. Data represent the average of 3 independent experiments ± SD.
(PDF)

S4 Fig DUB3 regulates the stability of Hippo proteins in BJ cells. (A) BJ fibroblast cells expressing hTert and H-Ras^G12V^ and depleted of p53 and p16 (BJ^p53kd/p16kd/HRas^) were virally transduced and selected to express DUB3, its inactive C89S mutant form or an empty control vector. Cells were plated for 36h before being harvested for immunoblotting. Blots were probed with antibodies against Flag, DUB3, ITCH, phospho-LATS1/2 (T1079/1041), LATS1, AMOT L1, phospho-YAP S127, YAP and actin. (B) BJ^p53kd/p16kd/HRas^ cells were virally transduced and selected to stably express either a control vector or independent shRNAs against DUB3 and processed as in (A). (C) BJ^p53kd/p16kd/HRas^ cells were transfected to express the luciferase reporters together with siRNAs to deplete DUB3 or with a control scrambled siRNA. Data represent the average of three independent transfection experiments ± SD.
(PDF)

S5 Fig YAP localization in BJ cells depleted of DUB3. (A) BJ^p53kd/p16kd/HRas^ cells were seeded for 24h before being transfected with DUB3-specific siRNAs or scrambled controls. Transfected cells were fixed after 36h and stained with YAP antibody (Santa Cruz, sc-101199). qPCR was used to confirm the depletion of DUB3. (B) Representative images of BJ cells stained with anti-YAP treated as described in panel A. YAP localization was scored as: less YAP in the nucleus compared to the cytoplasm (e.g. white arrows); equal in cytoplasm and nucleus (e.g. blue arrows); and YAP higher in the nucleus (e.g. red arrows). (C) DUB3 depletion caused a significant shift towards nuclear YAP (p<0,0001; Chi-square test).
(PDF)

S6 Fig Effect of DUB3 expression on NEDD4 E3 ligase family members. HEK293T cells were transfected with a vector expressing DUB3 or the inactive C89S mutant form or with an empty vector as a control. mRNA expression of NEDD4, ITCH and SMURF1 was measured by qPCR. GAPDH mRNA was used for normalization and TBP was used as an independent control. Data represent the average of 3 independent experiments ± SD.
(PDF)

S7 Fig Interaction between endogenous ITCH and DUB3. Immunoprecipitation assays. HEK293T cells were treated with 5μM MG132 to block proteasome function 24h before cells were harvested for IP by lysing in PLC buffer, or a modified RIPA buffer containing 10% glycerol. Lysates were immunoprecipitated with anti-ITCH or anti-DUB3 antibodies or isotype-matched control antibodies. Blots were probed with anti-ITCH, anti-DUB3 antibodies. Endogenous ITCH was recovered in the DUB3 IP, but DUB3 was not recovered in the ITCH IP.
(PDF)

S8 Fig DUB3 depletion increases ubiquitylation of ITCH. HEK293T cells were transfected to express Myc-tagged ITCH along with ubiquitin and siRNA targeting DUB3 or a scrambled control siRNA. Transfected cells were treated with 5μM of MG132 and 5μM of Lactacystin overnight before being subjected to immunoprecipitation with anti-Myc. Blots were probed with antibodies against DUB3, actin, and the Myc epitope to detect Myc-tagged ITCH and with antibodies specific to ubiquitin linked at lysine residues K48 and K63.
(PDF)

S9 Fig DUB3 affects ubiquitylation of AMOT protein. (A) HEK293T cells were transfected to express HA-tagged AMOT along with ubiquitin and DUB3 siRNA or a scrambled siRNA. Transfected cells were treated with 5μM of MG132 and 5μM of Lactacystin overnight before being subjected to immunoprecipitation with anti-HA. Blots were probed with antibodies against HA, DUB3, actin and specific lysine residues K48 and K63 of ubiquitin. (B) HEK293T cells were transfected to express HA-tagged AMOT together with ubiquitin and Flag-DUB3 or Flag-DUB3 C89S. Blots were probed with antibodies against Myc, HA, DUB3, actin.
(PDF)

S10 Fig DUB3 interacts with LATS2 protein and suppresses its ubiquitylation. (A) HEK293T cells were transfected to express Flag-DUB3, its inactive mutant C89S or a control vector. Transfected cells were treated with 5μM of MG132 overnight before being subjected to immunoprecipitation with anti-Flag. Blots were probed with antibodies against Flag, LATS2, HA and actin. (B) HEK293T cells were transfected to express HA-LATS2 and myc-ubiquitin along with Flag-DUB3, its inactive mutant C89S or a control vector. Cells were treated with 5μM of MG132 overnight before being subjected to immunoprecipitation with anti-HA. Blots were probed with antibodies against Myc, LATS2, HA, DUB3 and actin.
(PDF)

S1 Table Sequences of shRNA targets, siRNAs and qPCR primers. (PDF)

## Abbreviations

DUB: deubiquitylating enzyme
YAP: Yes-Associated Protein
TAZ: transcriptional coactivator with PDZ-binding motif
AMOT: angiomotin
LATS: large tumor suppressor
NEED4: neural precursor cell expressed developmentally down-regulated protein 4
