# Supplementary material for: DUB3 Deubiquitylating Enzymes Regulate Hippo Pathway Activity by Regulating the Stability of ITCH, LATS and AMOT Proteins
Source: PLoS One. 2017 Jan 6;12(1):e0169587. doi: 10.1371/journal.pone.0169587 (PMC5218808; doi:10.1371/journal.pone.0169587)
Supplement: S1 Fig — The NCBI RefSeq Gene collection comprises 30 USP17L (USP17L1 through USP17L30) gene entries: 25 are annotated as protein coding genes (each with an individual mRNA and protein RefSeq), while four are annotated as pseudogenes (USP17L6P, -9P, -14P, -16P), and one (USP17L23) is annotated as protein coding gene, but not linked to either a RefSeq mRNA or protein sequence. (http://www.ncbi.nlm.nih.gov/books/NBK21091). The table summarizes the number of mismatches between the individual siRNAs and shRNAs with the family members. (PDF) [file pone.0169587.s001.pdf]

Supplemental Figure S1. Summary of DUB3 family members

The NCBI RefSeq Gene collection comprises 30 USP17L (USP17L1 through USP17L30) gene entries: 25 are annotated as protein coding genes (each with an individual mRNA and protein RefSeq), while four are annotated as pseudogenes (USP17L6P, -9P, -14P, -16P), and one (USP17L23) is annotated as protein coding gene, but not linked to either a RefSeq mRNA or protein sequence. (http://www.ncbi.nlm.nih.gov/books/NBK21091)

The table summarizes the number of mismatches between the individual siRNAs and shRNAs with the family members

	siRNAs								shRNAs		
	Pool 1: J-027332				Pool 2: J190106						
	-11	-13	-24	-25	-05	-06	-07	-05	#1	#2	#3
**USP17L8**	0	1	0	1	3	1	1	1	2	0	0
**USP17L4**	0	1	0	1	3	1	1	1	2	0	1
**USP17L3**	0	0	0	1	3	1	2	1	2	0	1
**USP17L1**	0	0	0	1	3	3	2	1	2	0	1
**USP17L2**	0	0	0	0	3	1	1	1	2	0	0
**USP17L17**	0	1	1	1	1	1	0	0	0	0	0
**USP17L13**	1	1	1	1	2	2	0	0	0	0	0
**USP17L21**	1	1	1	1	1	0	0	0	0	0	0
**USP17L12**	1	1	1	1	1	0	0	0	0	1	0
**USP17L19**	1	1	1	1	0	0	0	0	0	1	0
**USP17L22**	1	1	1	1	1	0	0	0	0	1	0
**USP17L20**	1	0	1	1	1	0	0	0	0	0	0
**USP17L15**	1	0	1	1	1	0	0	0	1	0	0
**USP17L11**	1	0	1	1	1	0	0	0	0	0	0
**USP17L18**	1	0	1	1	0	0	0	0	0	0	0
**USP17L30**	0	1	1	1	2	1	0	0	0	0	0
**USP17L5**	0	1	1	1	2	1	0	0	0	0	0
**USP17L25**	0	1	1	1	2	1	0	0	0	0	0
**USP17L24**	0	1	1	1	2	1	0	0	0	0	0
**USP17L26**	0	1	1	1	2	1	0	0	0	0	0
**USP17L27**	0	1	1	1	2	1	0	0	0	0	0
**USP17L28**	0	1	1	1	2	1	0	0	0	0	0
**USP17L29**	0	1	1	1	2	1	0	0	0	0	0
**USP17L10**	1	0	1	2	2	2	0	0	1	0	0
**USP17L7**	1	0	2	2	3	3	1	1	2	0	1

Target sequences for the siRNAs and shRNAs used in this study are shown below.

(B) Pool J-027332 sequence 11

```
NM_001256872.1[USP17L8]     TGCCATGAAAAAGGCATGCCTTCCCGGCCACAAGCAGGTAGATCATCAC
NM_001256874.1[USP17L4]     TGCCATGAAAAAGGCATGCCTTCCCGGCCACAAGCAGGTAGATCATCAC
NM_001256871.1[USP17L3]     TGCCATGAAAAAGGCATGCCTTCCCGGCCACAAGCAGGTAGATCATCAC
NM_001256873.1[USP17L1]     TGCCATGAAAAAGGCATGCCTTCCCGGCCACAAGCAGGTAGATCATCAC
NM_201402.2[USP17L2]        TGCCATGAAAAAGGCATGCCTTCCCGGCCACAAGCAGGTAGATCATCAC
NM_001256857[USP17L17]      TGCCATGAAAAAGGCATGCCTTCCCGGGCACAAGCAGGTAGATCATCAC
NM_001256855.1[USP17L13]    TGCCATGAAAAAGGCATGCCTTCCCGGGCACAAGCAGGTAGATCATCCC
NM_001256862.1[USP17L21]    TGCCATGAAAAAGGCATGCCTTCCCGGGCACAAGCAGGTGGATCATCAC
NM_001256853.1[USP17L12]    TGCCATGAAAAAGGCATGCCTTCCCGGGCACAAGCAGGTGGATCATCAC
NM_001256860.1[USL17L19]    TGCCATGAAAAAGGCATGCCTTCCCGGGCACAAGCAGGTGGATCATCAC
NM_001256863.1[USP17L22]    TGCCATGAAAAAGGCATGCCTTCCCGGGCACAAGCAGGTGGATCATCAC
NM_001256861.1[USP17L20]    TGCCATGAAAAAGGCATGCCTTCCCGGGCACAAGCAGGTGGATCATCAC
NM_001256894.1[USP17L15]    TGCCATGAAAAAGGCATGCCTTCCCGGGCACAAGCAGGTGGATCATCAC
NM_001256854.1[USP17L11]    TGCCATGAAAAAGGCATGCCTTCCCGGGCACAAGCAGGTGGATCATCAC
NM_001256859.1[USP17L18]    TGCCATGAAAAAGGCATGCCTTCCCGGGCACAAGCAGGTGGATCATCAC
NM_001256867.1[USP17L30]    TGCCATGAAAAAGGCATGCCTTCCCGGGCACAAGCAGGTAGATCATCAC
NM_001242329.1[USP17L5]     TGCCATGAAAAAGGCATGCCTTCCCGGGCACAAGCAGGTAGATCATCAC
NM_001242326.1[USP17L25]    TGCCATGAAAAAGGCATGCCTTCCCGGGCACAAGCAGGTAGATCATCAC
NM_001242327.1[USP17L24]    TGCCATGAAAAAGGCATGCCTTCCCGGGCACAAGCAGGTAGATCATCAC
NM_001242328.1[USP17L26]    TGCCATGAAAAAGGCATGCCTTCCCGGGCACAAGCAGGTAGATCATCAC
NM_001242330.1[USP17L27]    TGCCATGAAAAAGGCATGCCTTCCCGGGCACAAGCAGGTAGATCATCAC
NM_001242331.1[USP17L28]    TGCCATGAAAAAGGCATGCCTTCCCGGGCACAAGCAGGTAGATCATCAC
NM_001242332.1[USP17L29]    TGCCATGAAAAAGGCATGCCTTCCCGGGCACAAGCAGGTAGATCATCAC
NM_001256852.1[USP17L10]    TGCCATGAGAAAGGCATGCCTTCCCGGGCACAAGCAGGTAGATCGTCAC
NM_001256869.1[USP17L7]     TGCCATGAAAAAGGCATGCCTTCCCGGGCACAAGCAGCTAGATCATCAC
J-027332-11                 -----------------------------ACAAGCAGGTAGATCATCA
                                                         ******** * **** **
```

(C) Pool J-027332 sequence 13

```
NM_001256872.1[USP17L8]     GCTCCCTGCTAAACCTCTCTTCGATGAACTCGACAGATCAGGAGTCCATG
NM_001256874.1[USP17L4]     GCTCCCTGCTAAACCTCTCTTCGATGAACTCGACAGATCAGGAGTCCATG
NM_001256871.1[USP17L3]     GCTCCCTGCTAAACCTCTCTTCGACGACCCGGACAGATCAGGAGTCCATG
NM_001256873.1[USP17L1]     GCTCCCTGCTAAACCTCTCTTCGACGACCCGGACAGATCAGGAGTCCATG
NM_201402.2[USP17L2]        GCTCCCTGCTAAACCTCTCTTCGACGACCCGGACAGATCAGGAGTCCGTG
NM_001256857[USP17L17]      GCTCCCTGCTAAACCTCTCTTCGTCGACCCCGACACATCAGGAGTCCATG
NM_001256855.1[USP17L13]    GCTCCCTGCTAAACCTCTCTTCGTCGACCCCGACACATCAGGAGTCCATG
NM_001256862.1[USP17L21]    GCTCCCTGCTAAACCTCTCTTCGTCGACCCCGACACATCAGGAGTCCATG
NM_001256853.1[USP17L12]    GCTCCCTGCTAAAACTCTCTTCGACGACCCCGACACATCAGGAGTCCATG
NM_001256860.1[USL17L19]    GCTCCCTGCTAAAACTCTCTTCGACGACCCCGACACATCAGGAGTCCATG
NM_001256863.1[USP17L22]    GCTCCCTGCTAAAACTCTCTTCGACGACCCCGACACATCAGGAGTCCATG
NM_001256861.1[USP17L20]    GCTCCCTGCTAAACCTCTCTTCGACGACCCCGACACATCAGGAGTCCATG
NM_001256894.1[USP17L15]    GCTCCCTGCTAAACCTCTCTTCGACGACCCCGACACATCAGGAGTCCATG
NM_001256854.1[USP17L11]    GCTCCCTGCTAAACCTCTCTTCGACGACCCCGACACATCAGGAGTCCATG
NM_001256859.1[USP17L18]    GCTCCCTGCTAAACCTCTCTTCGACGACCCCGACACATCAGGAGTCCATG
NM_001256867.1[USP17L30]    GCTCCCTGCTAAACCTCTCTTCGTCGACCCCGACACATCAGGAGTCCATG
NM_001242329.1[USP17L5]     GCTCCCTGCTAAACCTCTCTTCGTCGACCCCGACACATCAGGAGTCCATG
NM_001242326.1[USP17L25]    GCTCCCTGCTAAACCTCTCTTCGTCGACCCCGACACATCAGGAGTCCATG
NM_001242327.1[USP17L24]    GCTCCCTGCTAAACCTCTCTTCGTCGACCCCGACACATCAGGAGTCCATG
NM_001242328.1[USP17L26]    GCTCCCTGCTAAACCTCTCTTCGTCGACCCCGACACATCAGGAGTCCATG
NM_001242330.1[USP17L27]    GCTCCCTGCTAAACCTCTCTTCGTCGACCCCGACACATCAGGAGTCCATG
NM_001242331.1[USP17L28]    GCTCCCTGCTAAACCTCTCTTCGTCGACCCCGACACATCAGGAGTCCATG
NM_001242332.1[USP17L29]    GCTCCCTGCTAAACCTCTCTTCGTCGACCCCGACACATCAGGAGTCCATG
NM_001256852.1[USP17L10]    GCTCCCTGCTAAACCTCTCTTCGACGACCCCGACAGATCAGGAGTCCATG
NM_001256869.1[USP17L7]     GCTCCCTGCTAAACCTCTCTTCGACGAAACCGACAGATCAGGAGTCCATG
J-027332-13                 --------CTAAACCTCTCTTCGACGA-----------------------
                                    ***** *********   **
```

(D) Pool J-027332 sequence 24

```
NM_001256872.1[USP17L8]      ACACCGCCCCTTGCCAACTACATGCTGTCCCGGGAGCACTCTCAAACATG
NM_001256874.1[USP17L4]      ACACTGCCCCTTGCCAACTACATGCTGTCCCGGGAGCACTCTCAAACATG
NM_001256871.1[USP17L3]      ACACTGCCCCTTGCCAACTACATGCTGTCCCGGGAGCACTCTCAAACATG
NM_001256873.1[USP17L1]      ACACTGCCCCTTGCCAACTACATGCTGTCCCGGGAGCACTCTCAAACATG
NM_201402.2[USP17L2]         ACACCGCCCCTTGCCAACTACATGCTGTCCCGGGAGCACTCTCAAACATG
NM_001256857[USP17L17]       ACACCGCCCCTTGCCAACTACATGCTGTCCCGGGAGCACTCTCAAACGTG
NM_001256855.1[USP17L13]     ACACCGCCCCTTGCCAACTACATGCTGTCCCGGGAGCACTCTCAAACGTG
NM_001256862.1[USP17L21]     ACACCGCCCCTTGCCAACTACATGCTGTCCCGGGAGCACTCTCAAACGTG
NM_001256853.1[USP17L12]     ACACCGCCCCTTGCCAACTACATGCTGTCCCGGGAGCACTCTCAAACGTG
NM_001256860.1[USL17L19]     ACACCGCCCCTTGCCAACTACATGCTGTCCCGGGAGCACTCTCAAACGTG
NM_001256863.1[USP17L22]     ACACCGCCCCTTGCCAACTACATGCTGTCCCGGGAGCACTCTCAAACGTG
NM_001256861.1[USP17L20]     ACACCGCCCCTTGCCAACTACATGCTGTCCCGGGAGCACTCTCAAACGTG
NM_001256894.1[USP17L15]     ACACCGCCCCTTGCCAACTACATGCTGTCCCGGGAGCACTCTCAAACGTG
NM_001256854.1[USP17L11]     ACACCGCCCCTTGCCAACTACATGCTGTCCCGGGAGCACTCTCAAACGTG
NM_001256859.1[USP17L18]     ACACCGCCCCTTGCCAACTACATGCTGTCCCGGGAGCACTCTCAAACGTG
NM_001242326.1[USP17L25]     ACACCGCCCCTTGCCAACTACATGCTGTCCCGGGAGCACTCTCAAACGTG
NM_001242327.1[USP17L24]     ACACCGCCCCTTGCCAACTACATGCTGTCCCGGGAGCACTCTCAAACGTG
NM_001242328.1[USP17L26]     ACACCGCCCCTTGCCAACTACATGCTGTCCCGGGAGCACTCTCAAACGTG
NM_001256867.1[USP17L30]     ACACCGCCCCTTGCCAACTACATGCTGTCCCGGGAGCACTCTCAAACGTG
NM_001242332.1[USP17L29]     ACACCGCCCCTTGCCAACTACATGCTGTCCCGGGAGCACTCTCAAACGTG
NM_001242331.1[USP17L28]     ACACCGCCCCTTGCCAACTACATGCTGTCCCGGGAGCACTCTCAAACGTG
NM_001242329.1[USP17L5]      ACACCGCCCCTTGCCAACTACATGCTGTCCCGGGAGCACTCTCAAACGTG
NM_001242330.1[USP17L27]     ACACCGCCCCTTGCCAACTACATGCTGTCCCGGGAGCACTCTCAAACGTG
NM_001256852.1[USP17L10]     AAACCGCCACTTGCCAACTACATGCTGTTCCGGGAGCACTCTCAAACGTG
NM_001256869.1[USP17L7]      ACACTGCCGCTTTCCAACTACATGCTGTCCCGGGAGGACTCTCAAACGTG
J-027332-24                  ------------------------------CGGGAGCACTCTCAAACAT-
                                                           ****** ********** *
```

(E) Pool J-027332 sequence 25

```
NM_001256872.1[USP17L8]      TCAATCAAAATACAAGTGTGGGATGAAAAACCACCATCCTGAACAGCAAA
NM_001256874.1[USP17L4]      TCAATCAAAATACAAGTGTGGGATGAAAAACCACCATCCTGAACAGCAAA
NM_001256871.1[USP17L3]      TCAATCAAAATACAAGTGTGGGATGAAAAACCATCATCCTGAACAGCAAA
NM_001256873.1[USP17L1]      TCAATCAAAATACAAGTGTGGGATGAAAAACCATCATCCTGAACAGCAAA
NM_201402.2[USP17L2]         TCAATCGAAATACAAGTGTGGGATGAAAAACCATCATCCTGAACAGCAAA
NM_001256857[USP17L17]       TCAATCAAAATACAAGTGTGGGATGAAGAACCATCATCCTGAACAGCAAA
NM_001256855.1[USP17L13]     TCAATCAAAATACAAGTGTGGGATGAAGAACCATCATCCTGAACAGCAAA
NM_001256862.1[USP17L21]     TCAATCAAAATACAAGTGTGGGATGAAGAACCATCATCCTGAACAGCAAA
NM_001256853.1[USP17L12]     TCAATCAAAATACAAGTGTGGGATGAAGAACCATCATCCTGAACAGCAAA
NM_001256860.1[USL17L19]     TCAATCAAAATACAAGTGTGGGATGAAGAACCATCATCCTGAACAGCAAA
NM_001256863.1[USP17L22]     TCAATCAAAATACAAGTGTGGGATGAAGAACCATCATCCTGAACAGCAAA
NM_001256861.1[USP17L20]     TCAATCAAAATACAAGTGTGGGATGAAGAACCATCATCCTGAACAGCAAA
NM_001256894.1[USP17L15]     TCAATCAAAATACAAGTGTGGGATGAAGAACCATCATCCTGAACAGCAAA
NM_001256854.1[USP17L11]     TCAATCAAAATACAAGTGTGGGATGAAGAACCATCATCCTGAACAGCAAA
NM_001256859.1[USP17L18]     TCAATCAAAATACAAGTGTGGGATGAAGAACCATCATCCTGAACAGCAAA
NM_001242326.1[USP17L25]     TCAATCAAAATACAAGTGTGGGATGAAGAACCATCATCCTGAACAGCAAA
NM_001242327.1[USP17L24]     TCAATCAAAATACAAGTGTGGGATGAAGAACCATCATCCTGAACAGCAAA
NM_001242328.1[USP17L26]     TCAATCAAAATACAAGTGTGGGATGAAGAACCATCATCCTGAACAGCAAA
NM_001256867.1[USP17L30]     TCAATCAAAATACAAGTGTGGGATGAAGAACCATCATCCTGAACAGCAAA
NM_001242332.1[USP17L29]     TCAATCAAAATACAAGTGTGGGATGAAGAACCATCATCCTGAACAGCAAA
NM_001242331.1[USP17L28]     TCAATCAAAATACAAGTGTGGGATGAAGAACCATCATCCTGAACAGCAAA
NM_001242329.1[USP17L5]      TCAATCAAAATACAAGTGTGGGATGAAGAACCATCATCCTGAACAGCAAA
NM_001242330.1[USP17L27]     TCAATCAAAATACAAGTGTGGGATGAAGAACCATCATCCTGAACAGCAAA
NM_001256852.1[USP17L10]     TCAATCAAAATACAAGTGTCGGATGAAGAACCATCATCCTGAACAGCAAA
NM_001256869.1[USP17L7]      TCAATCAAAATACAAGTGTGGTATGAAAAACCATCATCCTGAACAGCAAA
J-027332-25                  -----CGAAATACAAGTGTGGGAT--------------------------
                                  * ************ * **
```

(E) Pool 190106 sequence 05

```
NM_001256872.1[USP17L8]     TCAGCGTCCCAAGTGCTGCATGCTCTGTACTATGCAAGCTCACATCACAT
NM_001256874.1[USP17L4]     TCAGCGTCCCAAGTGCTGCATGCTCTGTACTATGCAAGCTCACATCACAT
NM_001256871.1[USP17L3]     TCAGCGTCCCAAGTGCTGCATGCTCTGTACTATGCAAGCTCACATCACAT
NM_001256873.1[USP17L1]     TCAGCGTCCCAAGTGCTGCATGCTCTGTACTATGCAAGCTCACATCACAT
NM_201402.2[USP17L2]        TCAGCGTCCCAAGTGCTGCATGCTCTGTACTATGCAAGCTCACATCACAT
NM_001256857[USP17L17]      TCATCGTCACAAGGGCTGCATGCTCTGTACGATGCAAGCTCACATCACAC
NM_001256855.1[USP17L13]    TCATCGTCACAAGGGCTGCATGCTCTGTACTATGCAAGCTCACATCACAC
NM_001256862.1[USP17L21]    TCATCGTCACAAGGGCTGCATGCTCTGTACGATGCAAGCTCACATCACAC
NM_001256853.1[USP17L12]    TCATCGTCACAAGGGCTGCATGCTCTGTACGATGCAAGCTCACATCACAC
NM_001256860.1[USL17L19]    TCATCGTCACAAGGGCTGTATGCTCTGTACGATGCAAGCTCACATCACAC
NM_001256863.1[USP17L22]    TCATCGTCACAAGGGCTGCATGCTCTGTACGATGCAAGCTCACATCACAC
NM_001256861.1[USP17L20]    TCATCGTCACAAGGGCTGCATGCTCTGTACGATGCAAGCTCACATCACAC
NM_001256894.1[USP17L15]    TCATCGTCACAAGGGCTGCATGCTCTGTACGATGCAAGCTCACATCACAC
NM_001256854.1[USP17L11]    TCATCGTCACAAGGGCTGCATGCTCTGTACGATGCAAGCTCACATCACAC
NM_001256859.1[USP17L18]    TCATCGTCACAAGGGCTGTATGCTCTGTACGATGCAAGCTCACATCACAC
NM_001242326.1[USP17L25]    TCATCGTCACAAGGGCTGCATGCTCTGTACTATGCAAGCTCACATCACAC
NM_001242327.1[USP17L24]    TCATCGTCACAAGGGCTGCATGCTCTGTACTATGCAAGCTCACATCACAC
NM_001242328.1[USP17L26]    TCATCGTCACAAGGGCTGCATGCTCTGTACTATGCAAGCTCACATCACAC
NM_001256867.1[USP17L30]    TCATCGTCACAAGGGCTGCATGCTCTGTACTATGCAAGCTCACATCACAC
NM_001242332.1[USP17L29]    TCATCGTCACAAGGGCTGCATGCTCTGTACTATGCAAGCTCACATCACAC
NM_001242331.1[USP17L28]    TCATCGTCACAAGGGCTGCATGCTCTGTACTATGCAAGCTCACATCACAC
NM_001242329.1[USP17L5]     TCATCGTCACAAGGGCTGCATGCTCTGTACTATGCAAGCTCACATCACAC
NM_001242330.1[USP17L27]    TCATCGTCACAAGGGCTGCATGCTCTGTACTATGCAAGCTCACATCACAC
NM_001256852.1[USP17L10]    TCATCGTCACAAGGGCTGCATGCTCTGTACTATGCAAGCTCACATCACAA
NM_001256869.1[USP17L7]     TCATCTTCACAAGTGCTGCATGTTCTGTACTATGCAAGCTCACATCACAT
190106-05                   -------------GGCTGTATGCTCTGTACGA------------------
                                          **** *** ******* *
```

(F) Pool 190106 sequence 06

```
NM_001256872.1[USP17L8]     GCAGGTAGATCATCACTCTAAGGACACCACCCTCATCCACCAAA
NM_001256874.1[USP17L4]     GCAGGTAGATCATCACTCTAAGGACACCACCCTCATCCACCAAA
NM_001256871.1[USP17L3]     GCAGGTAGATCATCACTCTAAGGACACCACCCTCATCCACCAAA
NM_001256873.1[USP17L1]     GCAGGTAGATCATCACTGCAAGGACACCACCCTCATCCACCAAA
NM_201402.2[USP17L2]        GCAGGTAGATCATCACTCTAAGGACACCACCCTCATCCACCAAA
NM_001256857[USP17L17]      GCAGGTAGATCATCACTCTAAGGACACCACCCTCATCCACCAAA
NM_001256855.1[USP17L13]    GCAGGTAGATCATCCCTCTAAGGACACCACCCTCATCCACCAAA
NM_001256862.1[USP17L21]    GCAGGTGGATCATCACTCTAAGGACACCACCCTCATCCACCAAA
NM_001256853.1[USP17L12]    GCAGGTGGATCATCACTCTAAGGACACCACCCTCATCCACCAAA
NM_001256860.1[USL17L19]    GCAGGTGGATCATCACTCTAAGGACACCACCCTCATCCACCAAA
NM_001256863.1[USP17L22]    GCAGGTGGATCATCACTCTAAGGACACCACCCTCATCCACCAAA
NM_001256861.1[USP17L20]    GCAGGTGGATCATCACTCTAAGGACACCACCCTCATCCACCAAA
NM_001256894.1[USP17L15]    GCAGGTGGATCATCACTCTAAGGACACCACCCTCATCCACCAAA
NM_001256854.1[USP17L11]    GCAGGTGGATCATCACTCTAAGGACACCACCCTCATCCACCAAA
NM_001256859.1[USP17L18]    GCAGGTGGATCATCACTCTAAGGACACCACCCTCATCCACCAAA
NM_001242326.1[USP17L25]    GCAGGTAGATCATCACTCTAAGGACACCACCCTCATCCACCAAA
NM_001242327.1[USP17L24]    GCAGGTAGATCATCACTCTAAGGACACCACCCTCATCCACCAAA
NM_001242328.1[USP17L26]    GCAGGTAGATCATCACTCTAAGGACACCACCCTCATCCACCAAA
NM_001256867.1[USP17L30]    GCAGGTAGATCATCACTCTAAGGACACCACCCTCATCCACCAAA
NM_001242332.1[USP17L29]    GCAGGTAGATCATCACTCTAAGGACACCACCCTCATCCACCAAA
NM_001242331.1[USP17L28]    GCAGGTAGATCATCACTCTAAGGACACCACCCTCATCCACCAAA
NM_001242329.1[USP17L5]     GCAGGTAGATCATCACTCTAAGGACACCACCCTCATCCACCAAA
NM_001242330.1[USP17L27]    GCAGGTAGATCATCACTCTAAGGACACCACCCTCATCCACCAAA
NM_001256852.1[USP17L10]    GCAGGTAGATCGTCACTCTAAGGACACCACCCTCATCCACCAAA
NM_001256869.1[USP17L7]     GCAGCTAGATCATCACTCCAAGGACACCACCCTCATCCACCAAA
190106-06                   --AGGTGGATCATCACTCTAA----------------------
                              ** * **** ** **  **
```

(G) Pool 190106 sequence 07

```
NM_001256872.1[USP17L8]     AACGTCAGAAAAGTTGAAGGTACCCTGCCTCCCAACGTACTTGTGATTCATCAA
NM_001256874.1[USP17L4]     AACGTCAGAAAAGTTGAAGGTACCCTGCCTCCCAACGTACTTGTGATTCATCAA
NM_001256871.1[USP17L3]     AACGTCGGAAAAGTCGAAGGTACCCTGCCTCCCAACGCACTTGTGATTCATCAA
NM_001256873.1[USP17L1]     AACGTCGGAAAAGTCGAAGGTACCCTGCCTCCCAACGCACTTGTGATTCATCAA
NM_201402.2[USP17L2]        AACGTCAGAAAAGTCGAAGGTACCCTGCCTCCCAACGTACTTGTGATTCATCAA
NM_001256857[USP17L17]      AACGTCAGAAAAGTCGAAGGTACCCTGCCTCCCGACGTACTTGTGATTCATCAA
NM_001256855.1[USP17L13]    AACGTCAGAAAAGTCGAAGGTACCCTGCCTCCCGACGTACTTGTGATTCATCAA
NM_001256862.1[USP17L21]    AACGTCAGAAAAGTCGAAGGTACCCTGCCTCCCGACGTACTTGTGATTCATCAA
NM_001256853.1[USP17L12]    AACGTCAGAAAAGTCGAAGGTACCCTGCCTCCCGACGTACTTGTGATTCATCAA
NM_001256860.1[USL17L19]    AACGTCAGAAAAGTCGAAGGTACCCTGCCTCCCGACGTACTTGTGATTCATCAA
NM_001256863.1[USP17L22]    AACGTCAGAAAAGTCGAAGGTACCCTGCCTCCCGACGTACTTGTGATTCATCAA
NM_001256861.1[USP17L20]    AACGTCAGAAAAGTCGAAGGTACCCTGCCTCCCGACGTACTTGTGATTCATCAA
NM_001256894.1[USP17L15]    AACGTCAGAAAAGTCGAAGGTACCCTGCCTCCCGACGTACTTGTGATTCATCAA
NM_001256854.1[USP17L11]    AACGTCAGAAAAGTCGAAGGTACCCTGCCTCCCGACGTACTTGTGATTCATCAA
NM_001256859.1[USP17L18]    AACGTCAGAAAAGTCGAAGGTACCCTGCCTCCCGACGTACTTGTGATTCATCAA
NM_001242326.1[USP17L25]    AACGTCAGAAAAGTCGAAGGTACCCTGCCTCCCGACGTACTTGTGATTCATCAA
NM_001242327.1[USP17L24]    AACGTCAGAAAAGTCGAAGGTACCCTGCCTCCCGACGTACTTGTGATTCATCAA
NM_001242328.1[USP17L26]    AACGTCAGAAAAGTCGAAGGTACCCTGCCTCCCGACGTACTTGTGATTCATCAA
NM_001256867.1[USP17L30]    AACGTCAGAAAAGTCGAAGGTACCCTGCCTCCCGACGTACTTGTGATTCATCAA
NM_001242332.1[USP17L29]    AACGTCAGAAAAGTCGAAGGTACCCTGCCTCCCGACGTACTTGTGATTCATCAA
NM_001242331.1[USP17L28]    AACGTCAGAAAAGTCGAAGGTACCCTGCCTCCCGACGTACTTGTGATTCATCAA
NM_001242329.1[USP17L5]     AACGTCAGAAAAGTCGAAGGTACCCTGCCTCCCGACGTACTTGTGATTCATCAA
NM_001242330.1[USP17L27]    AACGTCAGAAAAGTCGAAGGTACCCTGCCTCCCGACGTACTTGTGATTCATCAA
NM_001256852.1[USP17L10]    AACGTCAGAAGAGTCGAAGGTACGGTGCCTCCCGACGTACTTGTGATTCATCAA
NM_001256869.1[USP17L7]     AACGTCAGAAAAGTTGAAGGTACCCTGCCTCCCAACGTACTTGTGATTCATCAA
190106-07                   --------------------------------CGACGTACTTGTGATTCAT---
                                                            * *** *************
```

(H) Pool 190106 sequence 08

```
NM_001256872.1[USP17L8]     GTGTCAAGCAAGCTTTGGAACAGTTGGTGAAGCCCGAAGAACTCAATGGAGAGAAT
NM_001256874.1[USP17L4]     GTGTCAAGCAAGCTTTGGAACAGTTGGTGAAGCCCGAAGAACTCAATGGAGAGAAT
NM_001256871.1[USP17L3]     GTGTCAAGCAAGCTTTGGAACAGTTGGTGAAGCCCGAAGAACTCAATGGAGAGAAT
NM_001256873.1[USP17L1]     GTGTCAAGCAAGCTTTGGAACAGTTGGTGAAGCCCGAAGAACTCAATGGAGAGAAT
NM_201402.2[USP17L2]        GTGTCAAGCAAGCTTTGGAACAGTTGGTGAAGCCCGAAGAACTCAATGGAGAGAAT
NM_001256857[USP17L17]      GTGTCCAGCAAGCTTTGGAACAGTTGGTGAAGCCCGAAGAACTCAATGGAGAGAAT
NM_001256855.1[USP17L13]    GTGTCCAGCAAGCTTTGGAACAGTTGGTGAAGCCCGAAGAACTCAATGGAGAGAAT
NM_001256862.1[USP17L21]    GTGTCCAGCAAGCTTTGGAACAGTTGGTGAAGCCCGAAGAACTCAATGGAGAGAAT
NM_001256853.1[USP17L12]    GTGTCCAGCAAGCTTTGGAACAGTTGGTGAAGCCCGAAGAACTCAATGGAGAGAAT
NM_001256860.1[USL17L19]    GTGTCCAGCAAGCTTTGGAACAGTTGGTGAAGCCCGAAGAACTCAATGGAGAGAAT
NM_001256863.1[USP17L22]    GTGTCCAGCAAGCTTTGGAACAGTTGGTGAAGCCCGAAGAACTCAATGGAGAGAAT
NM_001256861.1[USP17L20]    GTGTCCAGCAAGCTTTGGAACAGTTGGTGAAGCCCGAAGAACTCAATGGAGAGAAT
NM_001256894.1[USP17L15]    GTGTCCAGCAAGCTTTGGAACAGTTGGTGAAGCCCGAAGAACTCAATGGAGAGAAT
NM_001256854.1[USP17L11]    GTGTCCAGCAAGCTTTGGAACAGTTGGTGAAGCCCGAAGAACTCAATGGAGAGAAT
NM_001256859.1[USP17L18]    GTGTCCAGCAAGCTTTGGAACAGTTGGTGAAGCCCGAAGAACTCAATGGAGAGAAT
NM_001242326.1[USP17L25]    GTGTCCAGCAAGCTTTGGAACAGTTGGTGAAGCCCGAAGAACTCAATGGAGAGAAT
NM_001242327.1[USP17L24]    GTGTCCAGCAAGCTTTGGAACAGTTGGTGAAGCCCGAAGAACTCAATGGAGAGAAT
NM_001242328.1[USP17L26]    GTGTCCAGCAAGCTTTGGAACAGTTGGTGAAGCCCGAAGAACTCAATGGAGAGAAT
NM_001256867.1[USP17L30]    GTGTCCAGCAAGCTTTGGAACAGTTGGTGAAGCCCGAAGAACTCAATGGAGAGAAT
NM_001242332.1[USP17L29]    GTGTCCAGCAAGCTTTGGAACAGTTGGTGAAGCCCGAAGAACTCAATGGAGAGAAT
NM_001242331.1[USP17L28]    GTGTCCAGCAAGCTTTGGAACAGTTGGTGAAGCCCGAAGAACTCAATGGAGAGAAT
NM_001242329.1[USP17L5]     GTGTCCAGCAAGCTTTGGAACAGTTGGCGAAGCCCGAAGAACTCAATGGAGAGAAT
NM_001242330.1[USP17L27]    GTGTCCAGCAAGCTTTGGAACAGTTGGTGAAGCCCGAAGAACTCAATGGAGAGAAT
NM_001256852.1[USP17L10]    GTGTCCAGCAAGCTTTGGAACAGTTGGTGAAGCCCGAAGAACTCAATGGAGAGAAT
NM_001256869.1[USP17L7]     GTGTCAAGCAAGCTTTGGAACAGTTGGTGAAGCCCAAAGAACTCAATGGAGAGAAT
190106-08                   ----CCAGCAAGCTTTGGAACAG---------------------------------
                                 ****************
```

(I) shRNAs 1-3

```
NM_001256872.1[USP17L8]     GCGATGTCACAGGCAACAAACTTGCCAAGAATGTGCAATATCCTGAGTGC
NM_001256874.1[USP17L4]     CCGATGTCGCAGGCAACAAACTTGCCAAGAATGTGCAATATCCTGAGTGC
NM_001256871.1[USP17L3]     CCGATGTCGCAGGCAACAAACTTGCCAAGAATGTGCAATATCCTGAGTGC
NM_001256873.1[USP17L1]     CCGATGTCGCAGGCAACAAACTTGCCAAGAATGTGCAATATCCTGAGTGC
NM_201402.2[USP17L2]        CCGATGTCACAGGCAACAAACTTGCCAAGAATGTGCAATATCCTGAGTGC
NM_001256857[USP17L17]      CCGATGTGACAGGCAACAAGATTGCCAAGAATGTGCAATATCCTGAGTGC
NM_001256855.1[USP17L13]    CCGATGTGACAGGCAACAAGATTGCCAAGAATGTGCAATATCCTGAGTGC
NM_001256862.1[USP17L21]    CCGATGTCACAGGCAACAAGATTGCCAAGAATGTGCAATATCCTGAGTGC
NM_001256853.1[USP17L12]    CCGATGTCACAGGCAACAAGATTGCCAAGAATGTGCAATATCCTGAGTGC
NM_001256860.1[USL17L19]    CCGATGTCACAGGCAACAAGATTGCCAAGAATGTGCAATATCCTGAGTGC
NM_001256863.1[USP17L22]    CCGATGTCACAGGCAACAAGATTGCCAAGAATGTGCAATATCCTGAGTGC
NM_001256861.1[USP17L20]    CCGATGTCACAGGCAACAAGATTGCCAAGAATGTGCAATATCCTGAGTGC
NM_001256894.1[USP17L15]    CCGATGTCACAGGCAACAAGATTGACAAGAATGTGCAATATCCTGAGTGC
NM_001256854.1[USP17L11]    CCGATGTCACAGGCAACAAGATTGCCAAGAATGTGCAATATCCTGAGTGC
NM_001256859.1[USP17L18]    CCGATGTCACAGGCAACAAGATTGCCAAGAATGTGCAATATCCTGAGTGC
NM_001242326.1[USP17L25]    CCGATGTCACAGGCAACAAGATTGCCAAGAATGTGCAATATCCTGAGTGC
NM_001242327.1[USP17L24]    CCGATGTCACAGGCAACAAGATTGCCAAGAATGTGCAATATCCTGAGTGC
NM_001242328.1[USP17L26]    CCGATGTCACAGGCAACAAGATTGCCAAGAATGTGCAATATCCTGAGTGC
NM_001256867.1[USP17L30]    CCGATGTCACAGGCAACAAGATTGCCAAGAATGTGCAATATCCTGAGTGC
NM_001242332.1[USP17L29]    CCGATGTCACAGGCAACAAGATTGCCAAGAATGTGCAATATCCTGAGTGC
NM_001242331.1[USP17L28]    CCGATGTCACAGGCAACAAGATTGCCAAGAATGTGCAATATCCTGAGTGC
NM_001242329.1[USP17L5]     CCGATGTCACAGGCAACAAGATTGCCAAGAATGTGCAATATCCTGAGTGC
NM_001242330.1[USP17L27]    CCGATGTCACAGGCAACAAGATTGCCAAGAATGTGCAATATCCTGAGTGC
NM_001256852.1[USP17L10]    CCGATGTCACAGGCAACAAAATTGCCAAGAATGTGCAATATCCTGAGTGC
NM_001256869.1[USP17L7]     CCGATGTCACAGGCAACAAACTTGCCAAGAATGTGCAATATCCTAAGTGC
shRNA_1                     -------------------GATTGCCAAGAATGTGCAA------------
                                                 *** *************

NM_001256872.1[USP17L8]     GCTCCCTGCTAAACCTCTCTTCGATGAACTCGACAGATCAGGAGTCCATG
NM_001256874.1[USP17L4]     GCTCCCTGCTAAACCTCTCTTCGATGAACTCGACAGATCAGGAGTCCATG
NM_001256871.1[USP17L3]     GCTCCCTGCTAAACCTCTCTTCGACGACCCGGACAGATCAGGAGTCCATG
NM_001256873.1[USP17L1]     GCTCCCTGCTAAACCTCTCTTCGACGACCCGGACAGATCAGGAGTCCATG
NM_201402.2[USP17L2]        GCTCCCTGCTAAACCTCTCTTCGACGACCCGGACAGATCAGGAGTCCGTG
NM_001256857[USP17L17]      GCTCCCTGCTAAACCTCTCTTCGTCGACCCCGACACATCAGGAGTCCATG
NM_001256855.1[USP17L13]    GCTCCCTGCTAAACCTCTCTTCGTCGACCCCGACACATCAGGAGTCCATG
NM_001256862.1[USP17L21]    GCTCCCTGCTAAACCTCTCTTCGTCGACCCCGACACATCAGGAGTCCATG
NM_001256853.1[USP17L12]    GCTCCCTGCTAAAACTCTCTTCGACGACCCCGACACATCAGGAGTCCATG
NM_001256860.1[USL17L19]    GCTCCCTGCTAAAACTCTCTTCGACGACCCCGACACATCAGGAGTCCATG
NM_001256863.1[USP17L22]    GCTCCCTGCTAAAACTCTCTTCGACGACCCCGACACATCAGGAGTCCATG
NM_001256861.1[USP17L20]    GCTCCCTGCTAAACCTCTCTTCGACGACCCCGACACATCAGGAGTCCATG
NM_001256894.1[USP17L15]    GCTCCCTGCTAAACCTCTCTTCGACGACCCCGACACATCAGGAGTCCATG
NM_001256854.1[USP17L11]    GCTCCCTGCTAAACCTCTCTTCGACGACCCCGACACATCAGGAGTCCATG
NM_001256859.1[USP17L18]    GCTCCCTGCTAAACCTCTCTTCGACGACCCCGACACATCAGGAGTCCATG
NM_001242326.1[USP17L25]    GCTCCCTGCTAAACCTCTCTTCGTCGACCCCGACACATCAGGAGTCCATG
NM_001242327.1[USP17L24]    GCTCCCTGCTAAACCTCTCTTCGTCGACCCCGACACATCAGGAGTCCATG
NM_001242328.1[USP17L26]    GCTCCCTGCTAAACCTCTCTTCGTCGACCCCGACACATCAGGAGTCCATG
NM_001256867.1[USP17L30]    GCTCCCTGCTAAACCTCTCTTCGTCGACCCCGACACATCAGGAGTCCATG
NM_001242332.1[USP17L29]    GCTCCCTGCTAAACCTCTCTTCGTCGACCCCGACACATCAGGAGTCCATG
NM_001242331.1[USP17L28]    GCTCCCTGCTAAACCTCTCTTCGTCGACCCCGACACATCAGGAGTCCATG
NM_001242329.1[USP17L5]     GCTCCCTGCTAAACCTCTCTTCGTCGACCCCGACACATCAGGAGTCCATG
NM_001242330.1[USP17L27]    GCTCCCTGCTAAACCTCTCTTCGTCGACCCCGACACATCAGGAGTCCATG
NM_001256852.1[USP17L10]    GCTCCCTGCTAAACCTCTCTTCGACGACCCCGACAGATCAGGAGTCCATG
NM_001256869.1[USP17L7]     GCTCCCTGCTAAACCTCTCTTCGACGAAACCGACAGATCAGGAGTCCATG
shRNA_2                     GCTCCCTGCTAAACCTCTC-------------------------------
                            ************* *****

NM_001256872.1[USP17L8]     GCTGGCTTCCATAGAGGCAAGCAGGAAGATGCCCATGAATTTCTCATGTT
NM_001256874.1[USP17L4]     GCTGGCTTCCATAGAGGCAAGCAGGAAGATGTCCATGAATTTCTCATGTT
NM_001256871.1[USP17L3]     TCTGGCTTCCATAGAGGCAAGCAGGAAGATGTCCATGAATTTCTCATGTT
NM_001256873.1[USP17L1]     GCTGGCTTCCATAGAGGCAAGCAGGAAGATGTCCATGAATTTCTCATGTT
NM_201402.2[USP17L2]        GCTGGCTTCCATAGAGGCAAGCAGGAAGATGCCCATGAATTTCTCATGTT
NM_001256857[USP17L17]      GCTGGCTTCCATAGAGGCAAGCAGGAAGATGCCCATGAATTTCTCATGTT
NM_001256855.1[USP17L13]    GCTGGCTTCCATAGAGGCAAGCAGGAAGATGCCCATGAATTTCTCATGTT
NM_001256862.1[USP17L21]    GCTGGCTTCCATAGAGGCAAGCAGGAAGATGCCCATGAATTTCTCATGTT
NM_001256853.1[USP17L12]    GCTGGCTTCCATAGAGGCAAGCAGGAAGATGCCCATGAATTTCTCATGTT
NM_001256860.1[USL17L19]    GCTGGCTTCCATAGAGGCAAGCAGGAAGATGCCCATGAATTTCTCATGTT
NM_001256863.1[USP17L22]    GCTGGCTTCCATAGAGGCAAGCAGGAAGATGCCCATGAATTTCTCATGTT
NM_001256861.1[USP17L20]    GCTGGCTTCCATAGAGGCAAGCAGGAAGATGCCCATGAATTTCTCATGTT
NM_001256894.1[USP17L15]    GCTGGCTTCCATAGAGGCAAGCAGGAAGATGCCCATGAATTTCTCATGTT
NM_001256854.1[USP17L11]    GCTGGCTTCCATAGAGGCAAGCAGGAAGATGCCCATGAATTTCTCATGTT
NM_001256859.1[USP17L18]    GCTGGCTTCCATAGAGGCAAGCAGGAAGATGCCCATGAATTTCTCATGTT
NM_001242326.1[USP17L25]    GCTGGCTTCCATAGAGGCAAGCAGGAAGATGCCCATGAATTTCTCATGTT
NM_001242327.1[USP17L24]    GCTGGCTTCCATAGAGGCAAGCAGGAAGATGCCCATGAATTTCTCATGTT
NM_001242328.1[USP17L26]    GCTGGCTTCCATAGAGGCAAGCAGGAAGATGCCCATGAATTTCTCATGTT
NM_001256867.1[USP17L30]    GCTGGCTTCCATAGAGGCAAGCAGGAAGATGCCCATGAATTTCTCATGTT
NM_001242332.1[USP17L29]    GCTGGCTTCCATAGAGGCAAGCAGGAAGATGCCCATGAATTTCTCATGTT
NM_001242331.1[USP17L28]    GCTGGCTTCCATAGAGGCAAGCAGGAAGATGCCCATGAATTTCTCATGTT
NM_001242329.1[USP17L5]     GCTGGCTTCCATAGAGGCAAGCAGGAAGATGCCCATGAATTTCTCATGTT
NM_001242330.1[USP17L27]    GCTGGCTTCCATAGAGGCAAGCAGGAAGATGCCCATGAATTTCTCATGTT
NM_001256852.1[USP17L10]    GCTGGCTTCCATAGAGGCAAGCAGGAAGATGCCCATGAATTTCTCATGTT
NM_001256869.1[USP17L7]     GCTGGCTTCCATAGAGGTGAGCAGGAGGATGCCCATGAATTTCTCATGTT
shRNA3                      --------------------GCAGGAAGATGCCCATGAA-----------
                                                ****** **** *******
```
