# Supplementary material for: DUB3 Deubiquitylating Enzymes Regulate Hippo Pathway Activity by Regulating the Stability of ITCH, LATS and AMOT Proteins
Source: PLoS One. 2017 Jan 6;12(1):e0169587. doi: 10.1371/journal.pone.0169587 (PMC5218808; doi:10.1371/journal.pone.0169587)
Supplement: S2 Fig — Controls for the efficacy of shRNAs and siRNAs on DUB3 mRNA expression. (A) HEK293T cells were transfected with a control vector or with independent shRNAs targeting DUB3. (B) HEK293T cells were transfected with a control siRNA (scrambled sequence) or independent siRNAs targeting DUB3. Expression of DUB3 mRNA was measured by quantitative real-time RT-PCR (qPCR) using two independent pairs of primers. Primer pair #1 was used subsequently for measuring DUB3 expression. GAPDH mRNA was used for normalization. Data represent the average of 3 independent experiments ± SD. * indicates p <0.05 (Student’s T-test, 2-tailed unequal variance) comparing the test and control samples. (PDF) [file pone.0169587.s002.pdf]

Supplemental Figure S2. Effect of shRNAs and siRNAs on DUB3 mRNA expression.

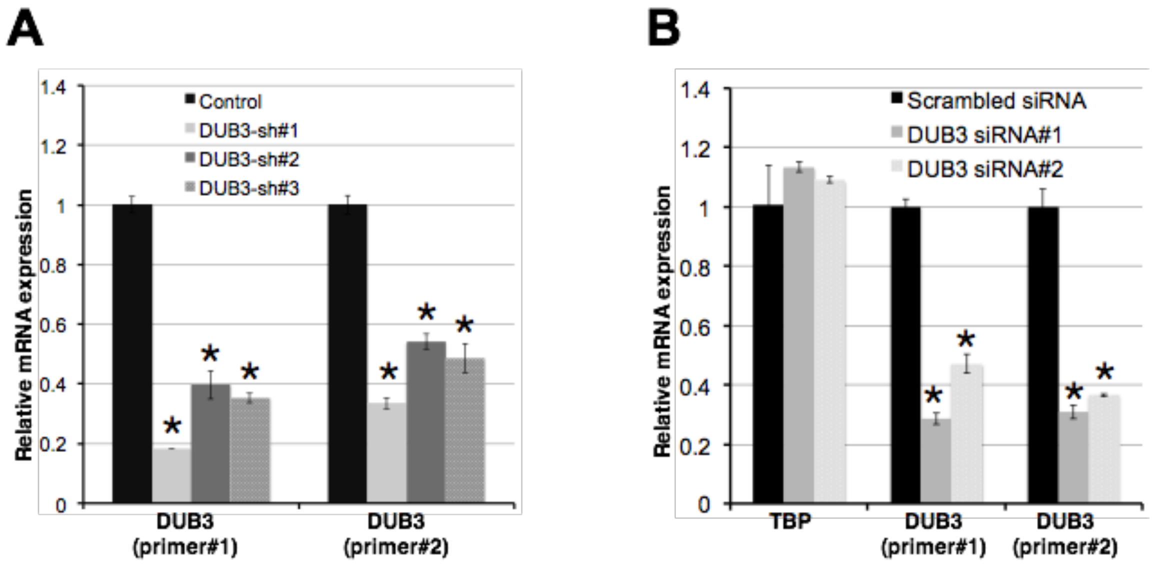

Controls for the efficacy of shRNAs and siRNAs on DUB3 mRNA expression. (**A)** HEK293T cells were transfected with a control vector or with independent shRNAs targeting DUB3. (**B**) HEK293T cells were transfected with a control siRNA (scrambled sequence) or independent siRNAs targeting DUB3. Expression of DUB3 mRNA was measured by quantitative real-time RT-PCR (qPCR) using two independent pairs of primers. Primer pair #1 was used subsequently for measuring DUB3 expression. GAPDH mRNA was used for normalization. Data represent the average of 3 independent experiments ± SD. * indicates $p < 0.05$ (Student's T-test, 2-tailed unequal variance) comparing the test and control samples.
