# Supplementary material for: DUB3 Deubiquitylating Enzymes Regulate Hippo Pathway Activity by Regulating the Stability of ITCH, LATS and AMOT Proteins
Source: PLoS One. 2017 Jan 6;12(1):e0169587. doi: 10.1371/journal.pone.0169587 (PMC5218808; doi:10.1371/journal.pone.0169587)
Supplement: S4 Fig — (A) BJ fibroblast cells expressing hTert and H-RasG12V and depleted of p53 and p16 (BJp53kd/p16kd/HRas) were virally transduced and selected to express DUB3, its inactive C89S mutant form or an empty control vector. Cells were plated for 36h before being harvested for immunoblotting. Blots were probed with antibodies against Flag, DUB3, ITCH, phospho-LATS1/2 (T1079/1041), LATS1, AMOT L1, phospho-YAP S127, YAP and actin. (B) BJp53kd/p16kd/HRas cells were virally transduced and selected to stably express either a control vector or independent shRNAs against DUB3 and processed as in (A). (C) BJp53kd/p16kd/HRas cells were transfected to express the luciferase reporters together with siRNAs to deplete DUB3 or with a control scrambled siRNA. Data represent the average of three independent transfection experiments ± SD. (PDF) [file pone.0169587.s004.pdf]

Supplemental Figure S4. DUB3 regulates the stability of Hippo proteins in BJ cells.

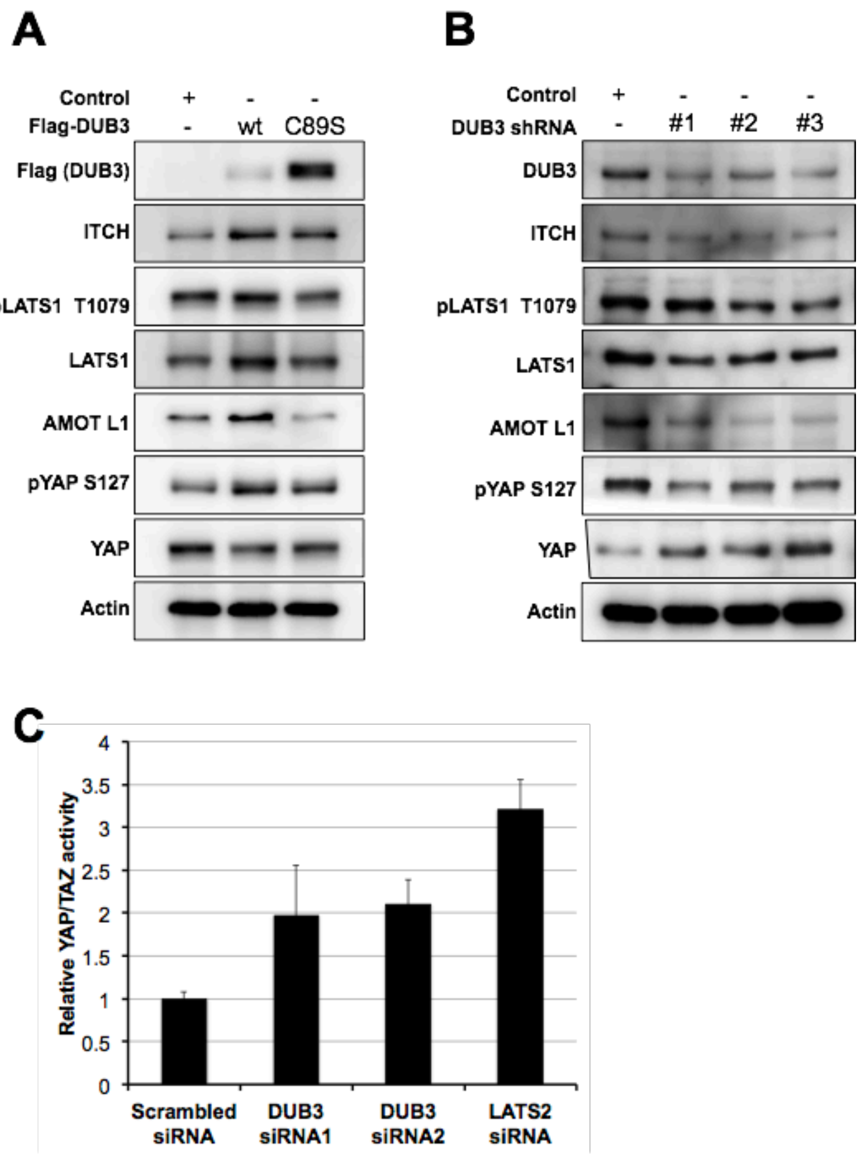

(A) BJ fibroblast cells expressing hTert and H-Ras^{G12V} and depleted of p53 and p16 (BJ$^{p53kd/p16kd/HRas}$) were virally transduced and selected to express DUB3, its inactive C89S mutant form or an empty control vector. Cells were plated for 36h before being harvested for immunoblotting. Blots were probed with antibodies against Flag, DUB3, ITCH, phospho-LATS1/2 (T1079/1041), LATS1, AMOT L1, phospho-YAP S127, YAP and actin.

(**B)** BJ$^{p53kd/p16kd/HRas}$ cells were virally transduced and selected to stably express either a control vector or independent shRNAs against DUB3 and processed as in (A).

(**C)** BJ$^{p53kd/p16kd/HRas}$ cells were transfected to express the luciferase reporters together with siRNAs to deplete DUB3 or with a control scrambled siRNA. Data represent the average of three independent transfection experiments ± SD.
