# Supplementary material for: DUB3 Deubiquitylating Enzymes Regulate Hippo Pathway Activity by Regulating the Stability of ITCH, LATS and AMOT Proteins
Source: PLoS One. 2017 Jan 6;12(1):e0169587. doi: 10.1371/journal.pone.0169587 (PMC5218808; doi:10.1371/journal.pone.0169587)
Supplement: S5 Fig — (A) BJp53kd/p16kd/HRas cells were seeded for 24h before being transfected with DUB3-specific siRNAs or scrambled controls. Transfected cells were fixed after 36h and stained with YAP antibody (Santa Cruz, sc-101199). qPCR was used to confirm the depletion of DUB3. (B) Representative images of BJ cells stained with anti-YAP treated as described in panel A. YAP localization was scored as: less YAP in the nucleus compared to the cytoplasm (e.g. white arrows); equal in cytoplasm and nucleus (e.g. blue arrows); and YAP higher in the nucleus (e.g. red arrows). (C) DUB3 depletion caused a significant shift towards nuclear YAP (p<0,0001; Chi-square test). (PDF) [file pone.0169587.s005.pdf]

Supplemental Figure S5. YAP localization in BJ cells depleted of DUB3

(A)

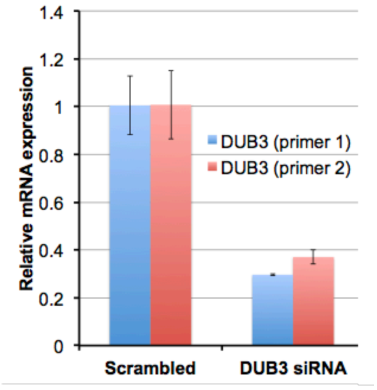

BJ$^{p53kd/p16kd/HRas}$ cells were seeded for 24h before being transfected with DUB3-specific siRNAs or scrambled controls. Transfected cells were fixed after 36h and stained with YAP antibody (Santa Cruz, sc-101199). qPCR was used to confirm the depletion of DUB3.

(B)

control cells DUB3-depleted cells

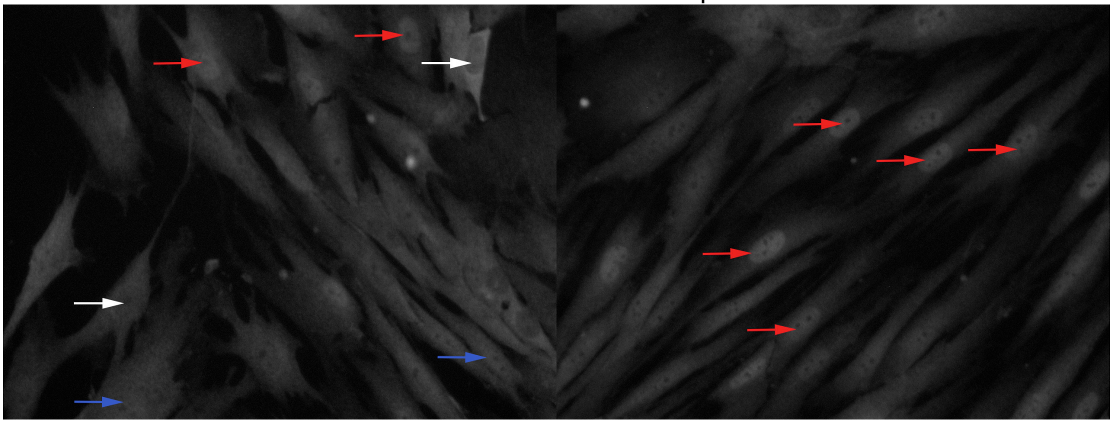

Representative images of BJ cells stained with anti-YAP treated as described in panel A. YAP localization was scored as: less YAP in the nucleus compared to the cytoplasm (e.g. white arrows); equal in cytoplasm and nucleus (e.g. blue arrows); and YAP higher in the nucleus (e.g. red arrows).

	number of cells		
	less nuclear YAP	even distribution	more nuclear YAP
control siRNA	118	120	84
DUB3 siRNA	54	239	177
	% of cells		
control siRNA	37	37	26
DUB3 siRNA	11	51	38

DUB3 depletion caused a significant shift towards nuclear YAP ($p<0,0001$; Chi-square test).
