# Supplementary material for: DUB3 Deubiquitylating Enzymes Regulate Hippo Pathway Activity by Regulating the Stability of ITCH, LATS and AMOT Proteins
Source: PLoS One. 2017 Jan 6;12(1):e0169587. doi: 10.1371/journal.pone.0169587 (PMC5218808; doi:10.1371/journal.pone.0169587)
Supplement: S6 Fig — HEK293T cells were transfected with a vector expressing DUB3 or the inactive C89S mutant form or with an empty vector as a control. mRNA expression of NEDD4, ITCH and SMURF1 was measured by qPCR. GAPDH mRNA was used for normalization and TBP was used as an independent control. Data represent the average of 3 independent experiments ± SD. (PDF) [file pone.0169587.s006.pdf]

Supplemental Figure S6: Effect of DUB3 expression on NEDD4 E3 ligase family members.

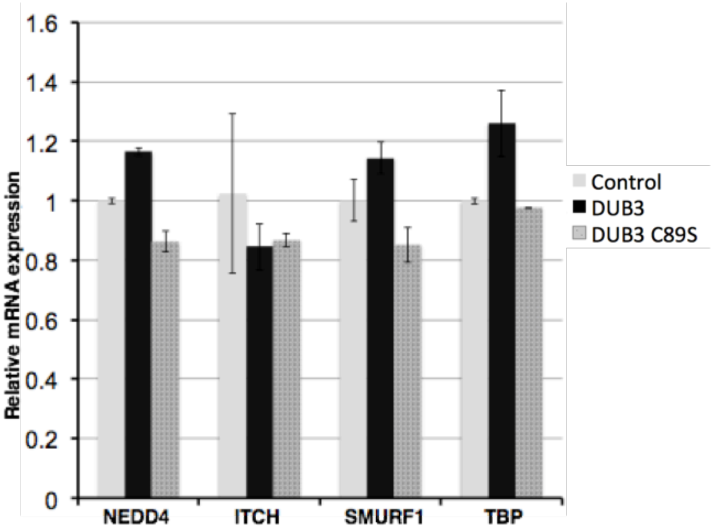

HEK293T cells were transfected with a vector expressing DUB3 or the inactive C89S mutant form or with an empty vector as a control. mRNA expression of NEDD4, ITCH and SMURF1 was measured by qPCR. GAPDH mRNA was used for normalization and TBP was used as an independent control. Data represent the average of 3 independent experiments ± SD.
