# Supplementary material for: DUB3 Deubiquitylating Enzymes Regulate Hippo Pathway Activity by Regulating the Stability of ITCH, LATS and AMOT Proteins
Source: PLoS One. 2017 Jan 6;12(1):e0169587. doi: 10.1371/journal.pone.0169587 (PMC5218808; doi:10.1371/journal.pone.0169587)
Supplement: S7 Fig — Immunoprecipitation assays. HEK293T cells were treated with 5μM MG132 to block proteasome function 24h before cells were harvested for IP by lysing in PLC buffer, or a modified RIPA buffer containing 10% glycerol. Lysates were immunoprecipitated with anti-ITCH or anti-DUB3 antibodies or isotype-matched control antibodies. Blots were probed with anti-ITCH, anti-DUB3 antibodies. Endogenous ITCH was recovered in the DUB3 IP, but DUB3 was not recovered in the ITCH IP. (PDF) [file pone.0169587.s007.pdf]

Supplemental Figure S7. Interaction between endogenous ITCH and DUB3.

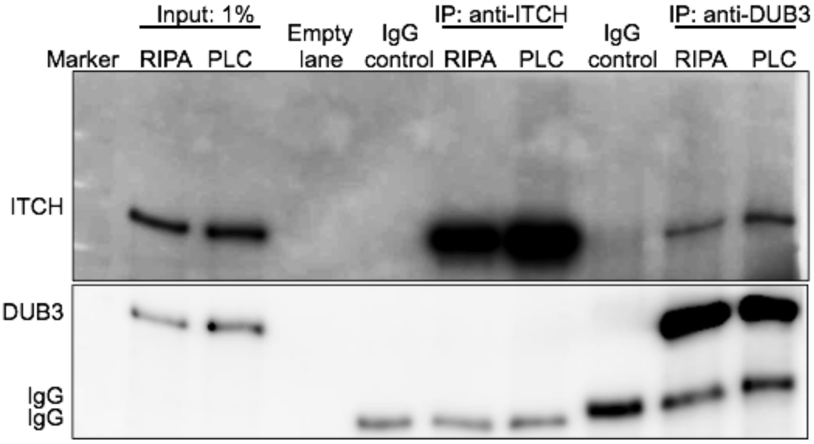

Immunoprecipitation assays. HEK293T cells were treated with 5μM MG132 to block proteasome function 24h before cells were harvested for IP by lysing in PLC buffer, or a modified RIPA buffer containing 10% glycerol. Lysates were immunoprecipitated with anti-ITCH or anti-DUB3 antibodies or isotype-matched control antibodies. Blots were probed with anti-ITCH, anti-DUB3 antibodies. Endogenous ITCH was recovered in the DUB3 IP, but DUB3 was not recovered in the ITCH IP.
