# Supplementary material for: DUB3 Deubiquitylating Enzymes Regulate Hippo Pathway Activity by Regulating the Stability of ITCH, LATS and AMOT Proteins
Source: PLoS One. 2017 Jan 6;12(1):e0169587. doi: 10.1371/journal.pone.0169587 (PMC5218808; doi:10.1371/journal.pone.0169587)
Supplement: S8 Fig — HEK293T cells were transfected to express Myc-tagged ITCH along with ubiquitin and siRNA targeting DUB3 or a scrambled control siRNA. Transfected cells were treated with 5μM of MG132 and 5μM of Lactacystin overnight before being subjected to immunoprecipitation with anti-Myc. Blots were probed with antibodies against DUB3, actin, and the Myc epitope to detect Myc-tagged ITCH and with antibodies specific to ubiquitin linked at lysine residues K48 and K63. (PDF) [file pone.0169587.s008.pdf]

Supplemental Figure S8. DUB3 depletion increases ubiquitylation of ITCH.

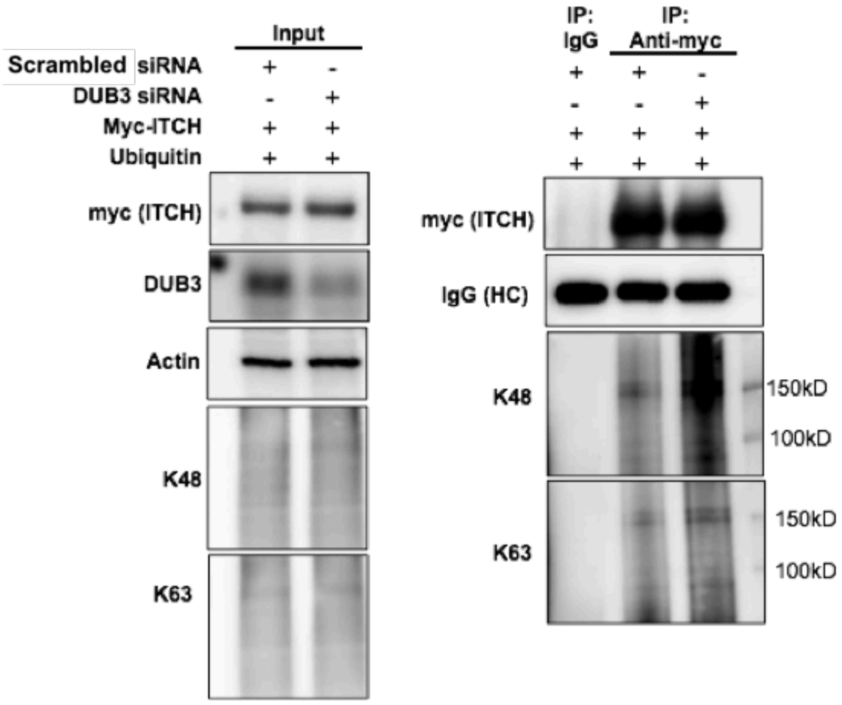

HEK293T cells were transfected to express Myc-tagged ITCH along with ubiquitin and siRNA targeting DUB3 or a scrambled control siRNA. Transfected cells were treated with 5μM of MG132 and 5μM of Lactacystin overnight before being subjected to immunoprecipitation with anti-Myc. Blots were probed with antibodies against DUB3, actin, and the Myc epitope to detect Myc-tagged ITCH and with antibodies specific to ubiquitin linked at lysine residues K48 and K63.
