# Supplementary material for: DUB3 Deubiquitylating Enzymes Regulate Hippo Pathway Activity by Regulating the Stability of ITCH, LATS and AMOT Proteins
Source: PLoS One. 2017 Jan 6;12(1):e0169587. doi: 10.1371/journal.pone.0169587 (PMC5218808; doi:10.1371/journal.pone.0169587)
Supplement: S9 Fig — (A) HEK293T cells were transfected to express HA-tagged AMOT along with ubiquitin and DUB3 siRNA or a scrambled siRNA. Transfected cells were treated with 5μM of MG132 and 5μM of Lactacystin overnight before being subjected to immunoprecipitation with anti-HA. Blots were probed with antibodies against HA, DUB3, actin and specific lysine residues K48 and K63 of ubiquitin. (B) HEK293T cells were transfected to express HA-tagged AMOT together with ubiquitin and Flag-DUB3 or Flag-DUB3 C89S. Blots were probed with antibodies against Myc, HA, DUB3, actin. (PDF) [file pone.0169587.s009.pdf]

Supplemental Figure S9. DUB3 affects ubiquitylation of AMOT protein.

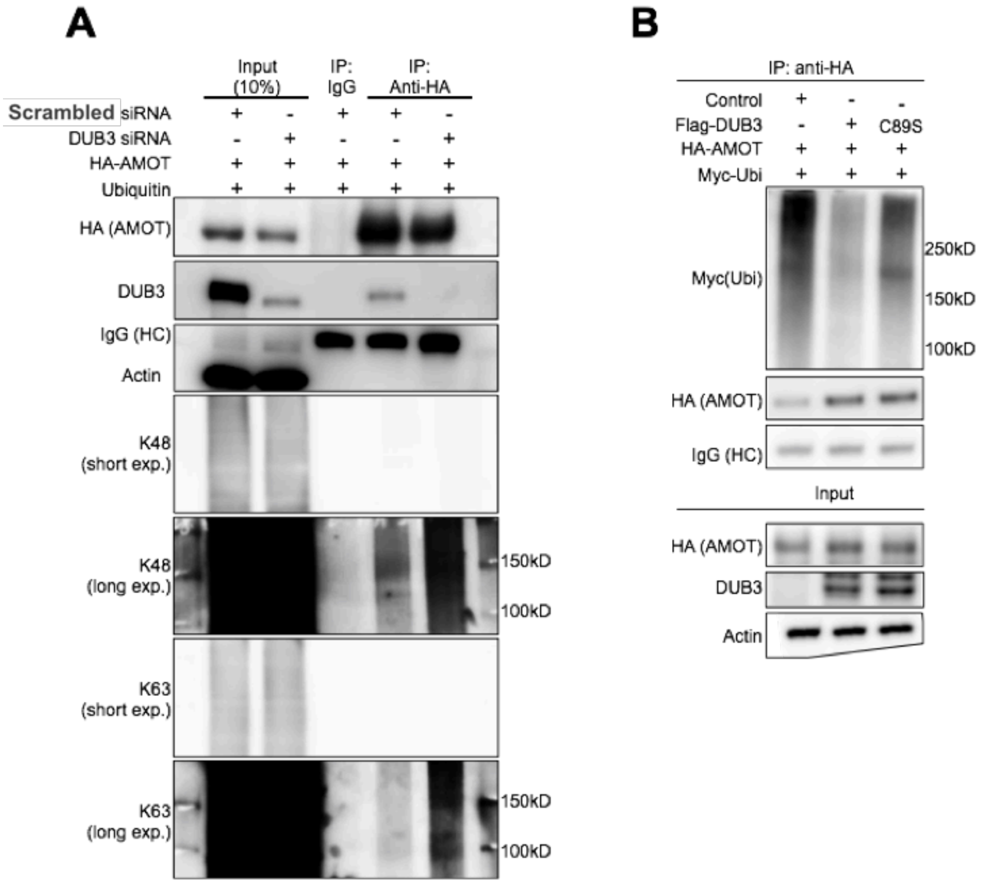

(**A**) HEK293T cells were transfected to express HA-tagged AMOT along with ubiquitin and DUB3 siRNA or a scrambled siRNA. Transfected cells were treated with 5μM of MG132 and 5μM of Lactacystin overnight before being subjected to immunoprecipitation with anti-HA. Blots were probed with antibodies against HA, DUB3, actin and specific lysine residues K48 and K63 of ubiquitin.
(**B**) HEK293T cells were transfected to express HA-tagged AMOT together with ubiquitin and Flag-DUB3 or Flag-DUB3 C89S. Blots were probed with antibodies against Myc, HA, DUB3, actin.
