# Supplementary material for: DUB3 Deubiquitylating Enzymes Regulate Hippo Pathway Activity by Regulating the Stability of ITCH, LATS and AMOT Proteins
Source: PLoS One. 2017 Jan 6;12(1):e0169587. doi: 10.1371/journal.pone.0169587 (PMC5218808; doi:10.1371/journal.pone.0169587)
Supplement: S10 Fig — (A) HEK293T cells were transfected to express Flag-DUB3, its inactive mutant C89S or a control vector. Transfected cells were treated with 5μM of MG132 overnight before being subjected to immunoprecipitation with anti-Flag. Blots were probed with antibodies against Flag, LATS2, HA and actin. (B) HEK293T cells were transfected to express HA-LATS2 and myc-ubiquitin along with Flag-DUB3, its inactive mutant C89S or a control vector. Cells were treated with 5μM of MG132 overnight before being subjected to immunoprecipitation with anti-HA. Blots were probed with antibodies against Myc, LATS2, HA, DUB3 and actin. (PDF) [file pone.0169587.s010.pdf]

Supplemental Figure S10. DUB3 interacts with LATS2 protein and suppresses its ubiquitylation.

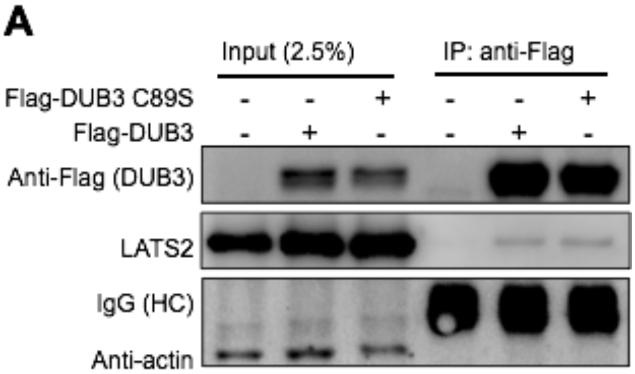

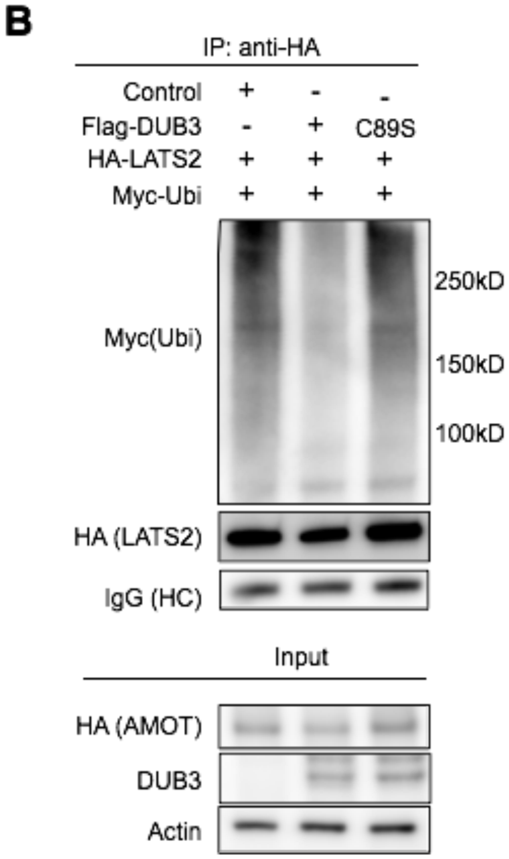

(**A**) HEK293T cells were transfected to express Flag-DUB3, its inactive mutant C89S or a control vector. Transfected cells were treated with 5μM of MG132 overnight before being subjected to immunoprecipitation with anti-Flag. Blots were probed with antibodies against Flag, LATS2, HA and actin.

(**B**) HEK293T cells were transfected to express HA-LATS2 and myc-ubiquitin along with Flag-DUB3, its inactive mutant C89S or a control vector. Cells were treated with 5μM of MG132 overnight before being subjected to immunoprecipitation with anti-HA. Blots were probed with antibodies against Myc, LATS2, HA, DUB3 and actin.
