# Supplementary material for: DUB3 Deubiquitylating Enzymes Regulate Hippo Pathway Activity by Regulating the Stability of ITCH, LATS and AMOT Proteins
Source: PLoS One. 2017 Jan 6;12(1):e0169587. doi: 10.1371/journal.pone.0169587 (PMC5218808; doi:10.1371/journal.pone.0169587)
Supplement: S1 Table — (PDF) [file pone.0169587.s011.pdf]

Table S1. Sequences of shRNA targets, siRNAs and qPCR primers

Gene	Sequence
DUB3	sh1 target sequence: GATTGCCAAGAATGTGCAA
	sh2 target sequence: GCTCCCTGCTAAACCTCTC
	sh3 target sequence: GCAGGAAGATGCCCATGAA
DUB3 siRNA Pool 1	J-027332-11: ACAAGCAGGTAGATCATCA
	J-027332-13: CTAAACCTCTCTTCGACGA
	J-027332-24: CGGGAGCACTCTCAAACAT
	J-027332-25: CGAAATACAAGTGTGGGAT
DUB3 siRNA Pool 2	190106-05: GGCTGTATGCTCTGTACGA
	190106-06: AGGTGGATCATCACTCTAA
	190106-07: CGACGTACTTGTGATTCAT
	190106-08: CCAGCAAGCTTTGGAACAG
DUB3 primer#1	Forward:5'-GCTCTTTCCACCAAGTGCTC-3'
	Reverse: 5'-CAGAAGACACAGACAGGCGA-3'
DUB3 primer#2	Forward:5'-CCTCCCGACGTACTTGTGAT-3'
	Reverse:5'-CATGGACTCCTGATGTGTCG-3'
TBP	Forward:5'-CGCCGAATATAATCCCAAGC-3'
	Reverse:5'-TCCTGTGCACACCATTTTCC-3'
LATS2	Forward:5'-TTGCTGATGTACTCCAGGGC-3'
	Reverse: 5'-AATGCTGCAGGAACTGGTG-3'
LATS1	Forward:5'-AGCTTGTGGTGGAATGTGAA-3'
	Reverse: 5'-TGGGACAACCTCCTTTCTTG-3'
YAP	Forward :5'-GCAACTCCAACCAGCAGCAACA-3'
	Reverse: 5'-CGCAGCCTCTCCTTCTCCATCTG-3'
TAZ	Forward :5'-ATTCATCGCCTTCCTAGGGT-3'
	Reverse: 5'-GGCTGGGAGATGACCTTCAC-3'
ITCH	Forward:5'-GAGGCTACCCATTGAACCAA-3'
	Reverse: 5'-AACGCCTTAACCCAAGGAAG-3'
AMOT	Forward:5'-CTTGATGGCCAATAAGCGTTGCCT -3'
	Reverse: 5'-GCAAGCCTGATCCAGCATTGGAAA -3'
NEDD4	Forward:5'-AAAGGGGGACCATCTCAGTT-3'
	Reverse: 5'-CCTTCAGGGAGCTGCTAAGA-3'
SMURF1	Forward:5'-AGACCCATCCACGACAATCT-3'
	Reverse: 5'-CAGCATCAAGATCCGTCTGA-3'
AMOT	Forward:5'-CTTGATGGCCAATAAGCGTTGCCT -3'
	Reverse: 5'-GCAAGCCTGATCCAGCATTGGAAA -3'
AMOT L1	Forward:5'-GGACAGGACTACTGGGGCTA-3'
	Reverse: 5'-GAACTAGCCATGATCGCCTC-3'
AMOT L2	Forward:5'-CATCTCTCGCTCCAGCTTCT-3'
	Reverse: 5'-CCTGGCAAGCAAGACACAG-3'
Cyr61	Forward: 5'-TATTCACAGGGTCTGCCCTC-3'
	Reverse: 5'-AACGAGGACTGCAGCAAAA-3'
ANKRD1	Forward: 5'-GTGTAGCACCAGATCCATCG-3'
	Reverse: 5'-CGGTGAGACTGAACCGCTAT-3'
